# Cyclic-di-GMP binds to histidine kinase RavS to control RavS-RavR phosphotransfer and regulates the bacterial lifestyle transition between virulence and swimming

**DOI:** 10.1371/journal.ppat.1007952

**Published:** 2019-08-13

**Authors:** Shou-Ting Cheng, Fang-Fang Wang, Wei Qian

**Affiliations:** 1 State Key Laboratory of Plant Genomics, Institution of Microbiology, Chinese Academy of Sciences, Beijing, China; 2 State Key Laboratory of Plant Genomics, Institution of Genetics and Development Biology, Chinese Academy of Sciences, Beijing, China; 3 College of Advanced Agricultural Sciences, University of Chinese Academy of Sciences, Beijing, China; 4 Center for Excellence in Biotic Interactions, Chinese Academy of Sciences, Beijing, China; University of Florida Institute of Food and Agricultural Sciences, UNITED STATES

## Abstract

The two-component signalling system (TCS) comprising a histidine kinase (HK) and a response regulator (RR) is the predominant bacterial sense-and-response machinery. Because bacterial cells usually encode a number of TCSs to adapt to various ecological niches, the specificity of a TCS is in the centre of regulation. Specificity of TCS is defined by the capability and velocity of phosphoryl transfer between a cognate HK and a RR. Here, we provide genetic, enzymology and structural data demonstrating that the second messenger cyclic-di-GMP physically and specifically binds to RavS, a HK of the phytopathogenic, gram-negative bacterium *Xanthomonas campestris* pv. *campestris*. The [c-di-GMP]-RavS interaction substantially promotes specificity between RavS and RavR, a GGDEF–EAL domain-containing RR, by reinforcing the kinetic preference of RavS to phosphorylate RavR. [c-di-GMP]-RavS binding effectively decreases the phosphorylation level of RavS and negatively regulates bacterial swimming. Intriguingly, the EAL domain of RavR counteracts the above regulation by degrading c-di-GMP and then increasing the level of phosphorylated RavS. Therefore, RavR acts as a bifunctional phosphate sink that finely controls the level of phosphorylated RavS. These biochemical processes interactively modulate the phosphoryl flux between RavS-RavR and bacterial lifestyle transition. Our results revealed that c-di-GMP acts as an allosteric effector to dynamically modulate specificity between HK and RR.

## Author summary

c-di-GMP is a multifunctional bacterial second messenger that controls various physiological processes. The nucleotide derivative binds to riboswitches or proteins as effectors during regulation. Here, we found that c-di-GMP physically binds to a histidine kinase, RavS, of a plant pathogenic bacterium. The binding event significantly enhanced the phosphotransferase activity of RavS to phosphorylate a response regulator, RavR. This process tightly modulates the phosphorylation level of RavS, which is important to the lifestyle transition of the bacterium between virulence and swimming motility. Therefore, our results reveal that c-di-GMP controls the bacterial two-component signalling, one of the dominant mechanisms of bacterial cells in adaptation to various environmental stimuli.

## Introduction

The two-component signalling system (TCS) is one of the predominant molecular machineries used by almost all bacteria to monitor and adaptively respond to environmental cues [1, 2]. The prototypical TCS is composed of a membrane-bound histidine kinase (HK) and a cytosolic response regulator (RR). Upon detecting a stimulus, HK autophosphorylates an invariant histidine residue within its dimerization and histidine phosphotransfer (DHp) domain and then catalyses the transfer of the phosphoryl group onto a conserved aspartic acid within the receiver (REC) domain of the cognate RR [3]. The activated RR then modulates bacterial adaptation by controlling gene transcription or cellular behaviour [4, 5]. There is a high level of specificity between a HK and its cognate RR, which is quantified by the kinetic preference during phosphotransfer [6, 7]. The complexity of TCS regulation was revealed after three decades of extensive investigations. Bacterial cells dynamically and elegantly regulate time, rhythm, space and flux of the phosphotransfer between the HK and RR to adapt to diverse ecological niches [8]. For example, hybrid-type HK-mediated phosphorelay, phosphatases, auxiliary proteins and small RNAs are involved in TCS regulation [9–13].

Cyclic di-GMP (c-di-GMP) is a ubiquitous second messenger involved in regulating bacterial physiology [14–17]. This signalling chemical interacts with riboswitches or protein as effectors to control a diverse set of processes, such as virulence, biofilm formation, motility, cell division and quorum-sensing [18–20]. The c-di-GMP effectors include the PilZ family of proteins, transcription factors, ATPases, transporters and various metabolic enzymes [15, 21, 22]. Intrinsically, the TCS is extensively involved in c-di-GMP signalling because approximately 6% of bacterial RRs encode three types of protein modules that control c-di-GMP turnover [18]. Among them, GGDEF domains have diguanylate cyclase activity to biosynthesize c-di-GMP from two GTP molecules [23], whereas EAL or HD-GYP domains contains phosphodiesterase activities to degrade c-di-GMP into pGpG or GMP, respectively [23–25]. Some RRs encoding degenerate GGDEF or EAL domains can act as c-di-GMP receptors [14, 26]. Recently, c-di-GMP was found to interact directly with CckA, an essential HK regulating cell division of *Caulobacter crescentus*, to inhibit kinase activity and activate phosphatase activity of CckA, which triggers DNA replication and initiation of the cell cycle [27–29]. This evidence indicates that c-di-GMP and TCS signalling are tightly associated. However, how c-di-GMP modulates the specificity between HKs and RRs and the biological significance of this signalling crosstalk remain unresolved.

The gram-negative bacterium *Xanthomonas campestris* pv. *campestris* is a model organism for studying plant pathology. This bacterium is the causative agent of black rot disease in various cruciferous plants and causes substantial loss of vegetable production yields worldwide [30]. The *X*. *campestris* pv. *campestris* genome encodes a large number of TCS proteins (approximately 52 HKs and 54 RRs) [31], and a number of these proteins, including RpfC-RpfG, RavS-RavR and PcrK-PcrR, are regulators of bacterial virulence that are associated with c-di-GMP turnover [32–36]. Among these regulators, RavS-RavR was reported to constitute a putative TCS. RavS is a membrane-bound HK with two tandem PAS domains as potential intracellular sensors, and RavR is a RR that contains N-terminal degenerate GGDEF and EAL domains [37]. In the vicinity of the *ravR*-*ravS* locus, a HK gene named *ravA* was identified and encodes a protein that may phosphorylate RavR [38]. Inactivation of *ravA* and *ravR* causes a significant decrease in virulence [37, 38]. Therefore, RavS-RavA-RavR is likely to form a “three-component signalling system” that most likely has complex associations and outputs. However, the regulatory relationship and protein phosphorylation processes among RavS-RavA-RavR, and the potential role of c-di-GMP in the dynamics of the signalling system, remain unknown.

In this study, we reveal that c-di-GMP regulates the HK-RR phosphotransfer flux. c-di-GMP physically binds to the catalytic and ATP-binding domain (CA) of RavS to significantly enhance the phosphotransferase activity of RavS towards RavR. This process efficiently decreases the phosphorylation level of RavS. In controlling bacterial swimming and flagellar development, the REC domain of RavR acts as a highly efficient phosphate sink to decrease the phosphorylated RavS level, which is subject to the control of c-di-GMP. Epistasis analysis also revealed that unlike typical TCSs, the RR RavR is upstream of the HK RavS in regulation. In addition, the EAL domain of RavR degrades c-di-GMP to reduce the RavS-RavR phosphotransfer, thereby maintaining a high level of phosphorylated RavS. Therefore, RavR subtly modulates the phosphorylation level of RavS, which is critical in controlling bacterial swimming and flagellar biogenesis. In this process, c-di-GMP controls the phosphotransfer flux from the HK to the RR and plays a critical role during the lifestyle transition between swimming and bacterial virulence.

## Results

### *ravA*-*ravR* and *ravS* control bacterial virulence, flagellar development and swimming motility

**Fig 1:**
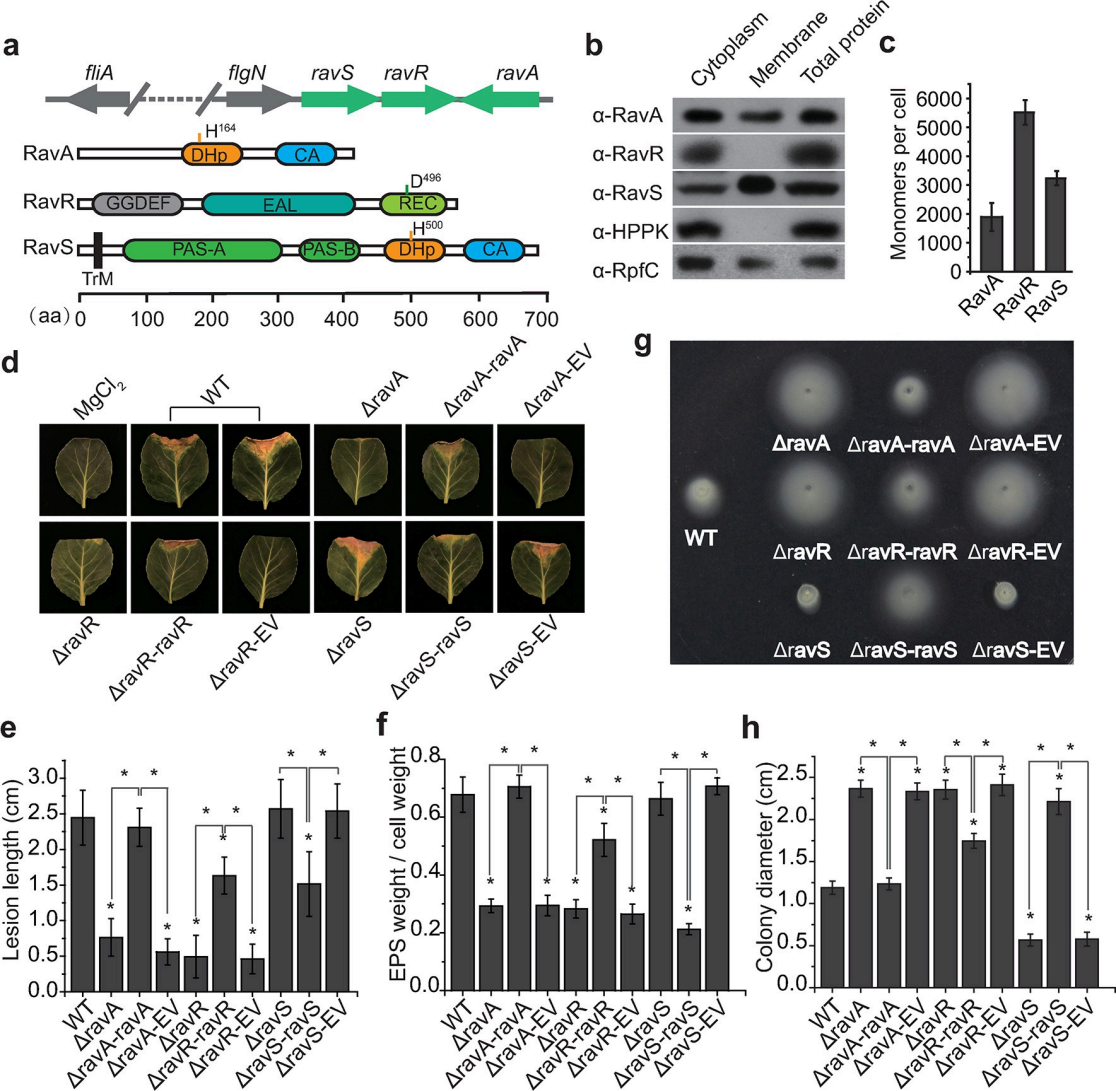
*ravA*, *ravR* and *ravS* differentially regulate bacterial virulence and swimming. (a) Genomic organisation of *ravARS* genes and putative secondary structures of their protein products. Protein secondary structures were predicted by the SMART program. TrM: transmembrane domain; PAS: Per-ARNT-Sim domain; DHp: dimerisation and histidine phosphotransfer domain; CA: catalytic and ATP binding domain; REC: receiver domain. GGDEF and EAL domains are also shown. (b) Subcellular localisation of RavA, RavR and RavS. Western blotting was used to detect the proteins in different subcellular fractions. The known membrane-bound RpfC and cytoplasmic HPPK were detected as controls. (c) The ratio of RavA, RavR and RavS proteins in bacterial cells. Semi-quantitative western blotting was used to estimate the levels of these proteins (*n* = 3). (b) and (c), each experiment was repeated three times. (d–e) Mutation of *ravA* and *ravR* rather than *ravS* attenuated bacterial virulence significantly. (d) Bacterial virulence against host plant *Brassica oleracea* cv Zhonggan 11. Strains were inoculated onto plant leaves by scissor cutting. The lesion length was recorded 10 d after inoculation. The negative control was an inoculation of 10 mM MgCl_2_. EV: empty vector. (e) Quantification of the lesion length in (d). Average lengths and standard deviations are shown. Asterisk: significant difference, as tested by Student’s *t*-test (*P* ≤ 0.05, *n* = 30 inoculation sites). (f) Extracellular polysaccharide (EPS) production of bacterial strains. EPS production was measured as the dry weight of EPS vs. the dry weight of bacterial cells. Asterisk: significant difference, as tested by Student’s *t*-test (*P* ≤ 0.05, *n* = 3). (g–h) Swimming motility of bacterial strains. (g) Bacterial strains were inoculated in NYG plates containing 0.15% agar and grown at 28°C for 28 h. (h) Average diameters of the migration zones of (g). Asterisk: significant difference, as tested by Student’s *t*-test (*P* ≤ 0.05, *n* = 10).

The genomic organization of *ravS*, *ravR* and *ravA* and the putative structures of their products are shown in Fig 1A. Western blotting revealed that RavS is located in the membrane and cytoplasm, and that RavR is a cytoplasmic protein (Fig 1B). Although RavA does not contain a recognisable transmembrane helix, repeated experiments showed that RavA is also located in both the membrane and cytoplasm (Fig 1B), which suggests that the HK has a membrane-anchored peptide or that the protein contains an unrecognised transmembrane region. Semi-quantitative western blotting demonstrated that the estimated ratio of RavA:RavR:RavS is approximately 10:29:17, amounting to approximately 1890, 5510 and 3230 molecules, respectively, in a bacterial cell grown under the tested conditions (Fig 1C and S1 Fig). The estimated concentrations of RavA, RavR and RavS in a cell were 2.05 ± 0.53, 5.99 ± 0.46, and 3.51 ± 0.27 μM, respectively (average bacterial cell volume was defined as 1.53 ± 0.23 μM^3^). This HK:RR ratio is remarkably higher than the general trend observed that RR molecules are often several dozen times more than the HK [39].

To further investigate the roles of *ravA*, *ravR* and *ravS* in controlling physiological processes, we performed phenotypic profiling of the in-frame deletion mutants of ΔravA, ΔravR and ΔravS. These mutants have phenotypes similar to those of the wild-type (WT) strain in growth in rich NYG medium, secretion of extracellular enzymes and resistance to oxidative stress (S2 Fig). However, these mutants exhibited remarkable but differing phenotypic changes in bacterial virulence and motility. Deletion of *ravA* and *ravR* substantially decreased bacterial virulence against the host plant *Brassica oleracea* cv. Zhonggan 11 (Fig 1D and 1E). Genetic complementation of the two genes, whose expression levels were controlled by their native promoters, fully or partially restored virulence to levels of the WT strain (Fig 1D and 1E). Although deletion of *ravS* has no impact on bacterial virulence, a genetic complementary strain (ΔravS-ravS) showed significant attenuation in virulence (Fig 1D and 1E), which might be caused by overexpression of *ravS* in a medium-copy, broad-host vector (pHM2). Accordingly, the production of extracellular polysaccharides (EPS), a major virulence determinate of *X*. *campestris* pv. *campestris*, decreased significantly to 43% and 42% of the levels of the WT strain in the ΔravA and ΔravR mutants, respectively, whereas the EPS levels were similar to the WT strain in their genetic complementary strains (Fig 1F). EPS production remained nearly unchanged in the *ravS* null mutant but decreased significantly in the *ravS* complementary strain (31% compared with that of the WT strain, (Fig 1F)). These results indicate that *ravA* and *ravR* are positive regulators of bacterial virulence but overexpression of *ravS* decreased bacterial virulence.

**Fig 2:**
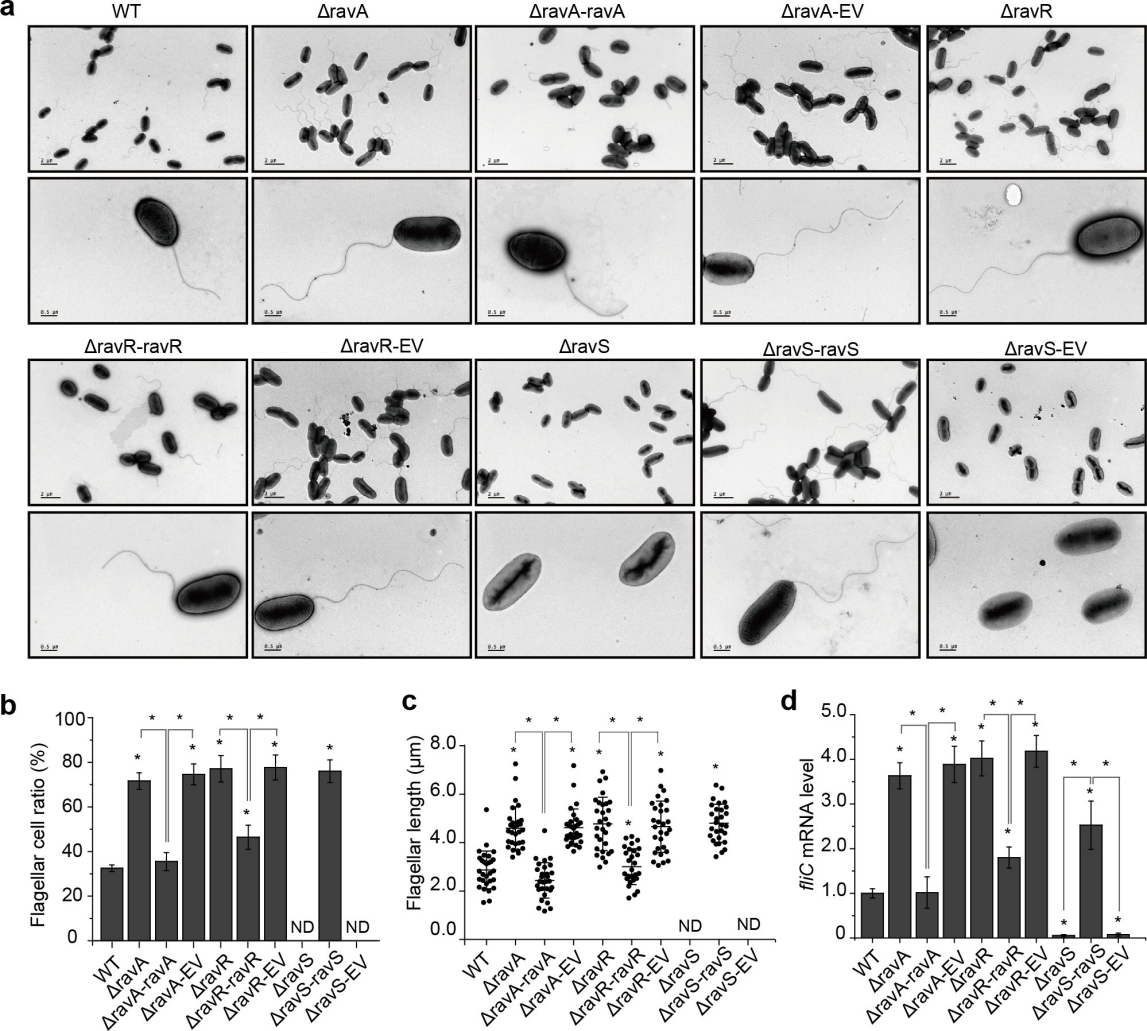
*ravA*, *ravR* and *ravS* differentially regulate the development of bacterial flagella. (a) Morphology of bacterial flagella. Bacterial flagella were observed by transmission electron microscopy after negative staining. A representative image of each strain is shown (*n* > 30). Upper panel: bacterial population profile; lower panel: morphology of a single bacterium. (b) Ratio of bacteria with flagella. For each strain, cells with flagella were counted for three biological replicates with each containing at least 100 cells. (c) The average flagellar length of bacterial strains. The length was measured using the AutoCAD software. Three biological replicates were taken with each comprising at least 30 measurements. (d) The *fliC* mRNA level in bacterial strains. The level of *fliC* mRNA was measured by qRT-PCR. Amplification of cDNA from tmRNA was used as an internal control. The experiment was repeated three times and the result of a representative experiment is shown. In (b–d), standard deviations are provided; asterisk: significant difference, as tested by Student’s *t*-test (*P* ≤ 0.05).

As shown in Fig 1G and 1H, compared with the WT, deletions of *ravA* and *ravR* increased swimming motility by approximately two-fold, but deletion of *ravS* almost completely eradicated the swimming capability of the mutant. Genetic complementation successfully restored the observed deficiencies to levels similar to that of WT strain (Fig 1G and 1H). Since bacterial swimming motility is controlled mainly by flagella, bacterial flagella were observed by transmission electron microscopy (TEM). As measured by the flagellar cell ratio (Fig 2A and 2B), *ravA* (flagellar cell ratio = 71.6%) and *ravR* (77.1%) mutant strains had a higher frequency of flagella in cells and significantly longer flagella than observed in the WT strain (32.5%). The average flagellar lengths of ΔravA and ΔravR mutant strains and the WT strain were 4.60, 4.84 and 2.82 μm (Fig 2C), respectively. However, inactivation of *ravS* resulted in complete loss of flagella (Fig 2A–2C). Genetic complementation of *ravA*, *ravR* or *ravS* in the corresponding mutant significantly suppressed these deficiencies in flagellar morphology.

*ravA*-*ravR*-*ravS* is located in the vicinity of gene clusters that encode flagella-associated proteins and may regulate the expression of these genes (S3 Fig). We selected eight genes to examine their transcription levels in various strains and found that *fliC* (encoding flagellin) is a representative gene that is controlled by *ravA*-*ravR*-*ravS* (S3A Fig). Deletion of *ravA* and *ravR* significantly increased the mRNA levels of *fliC* to 363% and 402% of the levels of the WT strain, respectively (Fig 2D). In contrast, mutation of *ravS* caused a significant decrease in the *fliC* transcription level to 5.6% of the level of the WT strain, while genetic complementation of *ravS* increased *fliC* mRNA to 253% of the level of the WT strain (Fig 2D). Based on these results, in controlling the development of bacterial flagella and *fliC* transcription, both *ravA* and *ravR* act as negative regulators but *ravS* is a positive regulator.

In addition, because type III secretion (T3SS) genes (*hrp*) are critical in virulence of *X*. *campestris*, we selected three *hrp* cluster genes (*hrpD*, *hrpF* and *hrpG*) to measure their expression levels. As shown in S3C Fig, the mRNA levels of *hrpF* did not have remarkable change in the *ravR* and *ravA* mutants. However, the amounts of *hrpG* and *hrpD* transcripts were significantly decreased comparing to the WT strain. Therefore, we further analysed the expression level of *hrpG* because HrpG is an OmpR-family response regulator to control the transcription of an AraC-family transcription factor HrpX, and the latter binds to the plant inducible promoters (PIP) of some *hrp* gene clusters to modulate the expressions of these T3SS genes [30, 40]. In the *ravR* and *ravA* mutants, the *hrpG* transcripts significantly decreased to the 5.2% and 2.2% levels of the WT strain, while genetic complementation of the two genes restored the *hrpG* expression to 111.1% and 75.6% levels of the WT strain (S3D Fig). This result reveals that *ravR* and *ravA* positively modulate the transcription of *hrpG*.

### *ravS* is genetically downstream of *ravA*–*ravR* in regulating bacterial swimming

**Fig 3:**
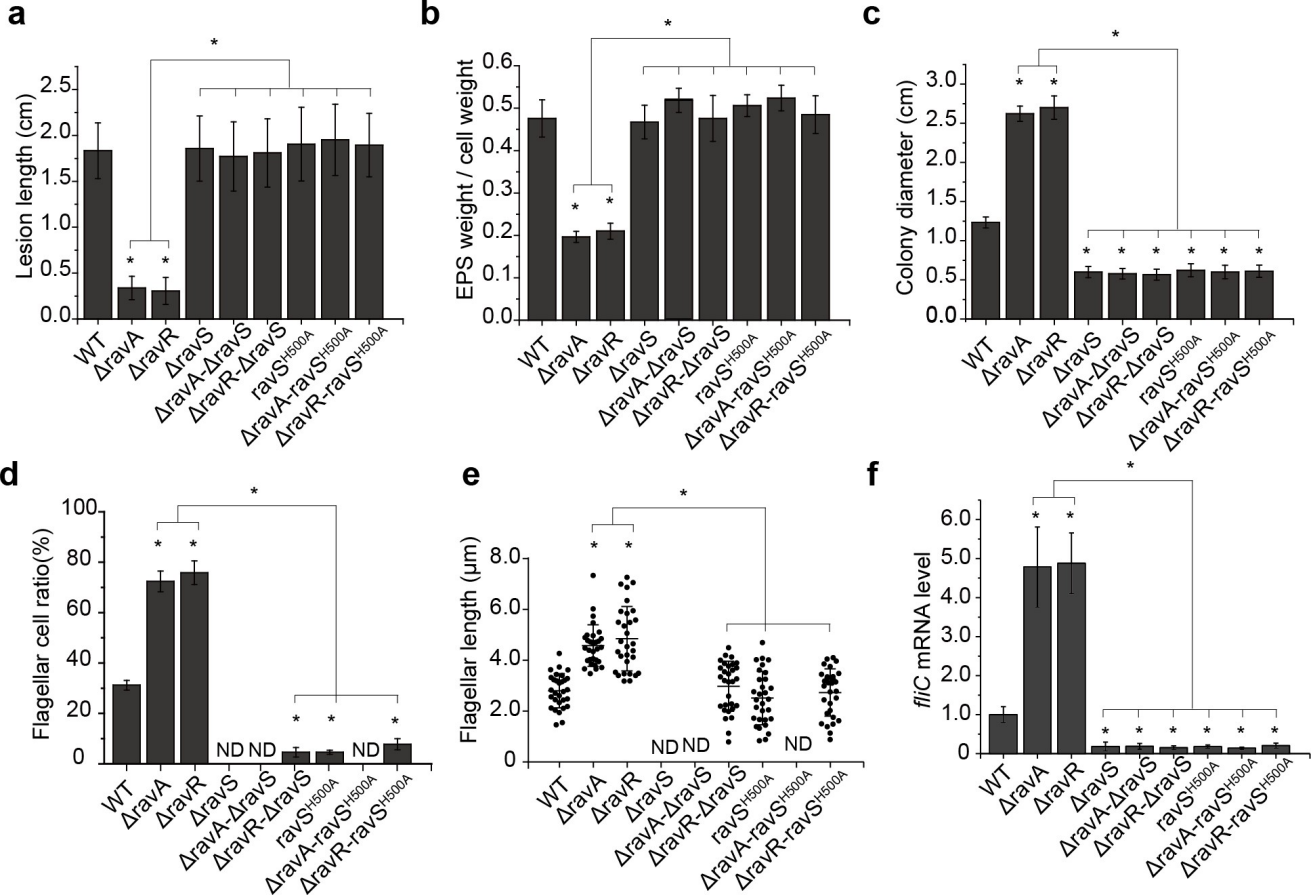
Epistatic analysis revealed that *ravS* is downstream of *ravA*-*ravR* in regulation. (a–f) Deletion of *ravS* or the ravS^H500A^ point mutation effectively suppressed the phenotypic deficiencies of *X*. *campestris* pv. *campestris* caused by *ravA* or *ravR* mutations. (a) Bacterial virulence measured by the lesion length on leaves. Bacterial strains were inoculated onto plant leaves of *Brassica oleracea* cv Zhonggan 11. The lesion length was recorded 10 d after inoculation (*n* = 30). (b) Production of extracellular polysaccharides (EPS). EPS production was quantified as the ratio of dry weight of EPS vs. the dry weight of bacterial cells (*n* = 3). (c) Bacterial swimming motility measured by the diameters of swimming zones. Bacterial strains were inoculated in NYG plates containing 0.15% agar and grown at 28°C for 28 h. The average diameters of the migration zones were measured (*n* = 10). (d) Ratio of bacterial cells with flagella. For each strain, cells with flagella were counted (*n* = 100). (e) The average flagellar length of bacterial strains (*n* = 30). (f) *fliC* mRNA levels in bacterial strains. The level of *fliC* mRNA was measured by qRT-PCR. Amplification of cDNA from tmRNA was used as an internal control. The experiment was repeated three times and the result of a representative experiment is shown. In (a–f), standard deviations are provided; asterisk: significant difference, as tested by Student’s *t*-test (*P* ≤ 0.05).

Epistatic analysis was performed to dissect the regulatory relationships among *ravR* and *ravA* or *ravS*. Although deletion of *ravA* or *ravR* significantly decreased the bacterial virulence and production of EPS (Fig 1), the double mutants (ΔravA-ΔravS or ΔravR-ΔravS) exhibited virulence levels similar to those of the WT strain (Fig 3A and 3B and S4A Fig). Similarly, inactivation of *ravS* effectively suppressed the increased swimming motility, flagellar cell ratio and flagellar length that were caused by *ravA* or *ravR* deletion (Fig 3C–3E, and S4B and S4C Fig). The *fliC* expression level of the *ravA* or *ravR* mutant was increased significantly (Fig 2D). However, in the double mutants, *fliC* mRNA levels decreased significantly to the level of ΔravS (Fig 3F). Furthermore, two double mutants on ΔravA and ΔravR backgrounds were constructed with the conserved phosphorylation residue (His^500^) of RavS substituted by Ala (ΔravA-ravS^H500A^ and ΔravR-ravS^H500A^). The phenotypic changes in the single mutant ravS^H500A^ were similar to those observed for the ΔravS mutant, and in the double mutants, analyses revealed that the *ravS*^H500A^ point mutation effectively suppressed all the tested phenotypic deficiencies that were caused by the *ravA* or *ravR* deletion (Fig 3A–3F and S4A–S4C Fig). Collectively, these genetic results suggest that *ravS* is downstream of *ravR* and *ravA* in regulation. The point mutant *ravS*^H500A^, in which RavS is constitutively dephosphorylated, exhibits reduced swimming capacity, which suggests that a high level of phosphorylated RavS (RavS~P) positively controls bacterial swimming but negatively modulates virulence. Furthermore, the suppressive function of *ravS* depends completely on the function of the conserved His^500^ residue, which suggests that RavA and RavR may negatively regulate the RavS~P level in the epistasis relationship.

### The EAL domain of RavR positively regulates RavS-mediated bacterial swimming in an Asp^496^-dependent manner

**Fig 4:**
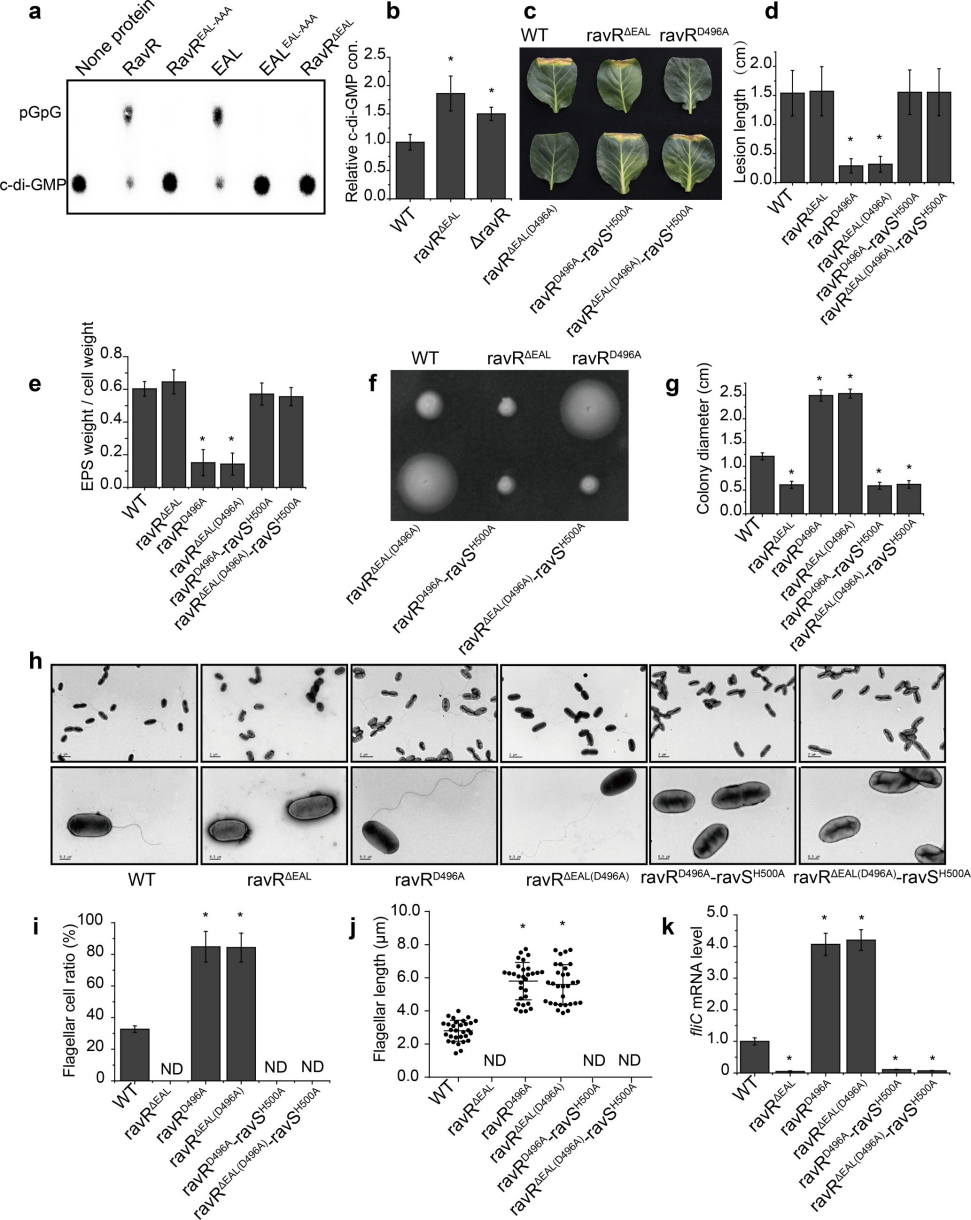
The EAL domain counteracts the regulation of phosphorylation residue Asp^496^ of RavR. (a) RavR and its EAL domain have phosphodiesterase activities to degrade c-di-GMP. ^32^P-labelled c-di-GMP was synthesised by tDGC cyclase *in vitro*. None or 5 μM purified proteins were added to the PDE reaction mixture with 1 μM ^32^P-labelled c-di-GMP, and reactions were incubated at 28°C for 30 min. Samples were analysed on thin-layer chromatography plates. (b) Deletion of coding sequences of the EAL domain and *ravR* increased intracellular c-di-GMP concentrations. Cellular c-di-GMP was extracted from 5 mL mid-log phase bacterial cells (OD_600_ = 0.8) and quantified by HPLC-MS/MS. (a, b) Each experiment was conducted three times. (c, d) Bacterial virulence assay. (c) Bacterial strains were inoculated onto plant leaves of *Brassica oleracea* cv Zhonggan 11. (d) The lesion length was recorded 10 d after inoculation (*n* = 30). (e) Production of extracellular polysaccharides (EPS). Bacterial strains were grown in TGM medium at 28°C for 72 h before EPS quantification, which was calculated as the dry weight of EPS vs. the dry weight of bacterial cells (*n* = 3). (f, g) Bacterial swimming motility. (f) Bacterial strains were inoculated in NYG plates containing 0.15% agar and grown at 28°C for 28 h. (g) Average diameters of the migration zones were measured (*n* = 10). (h–j) Morphology of bacterial flagella. (h) Bacterial flagella were observed by TEM. A representative image of each strain is shown. Upper panel: bacterial population profile; lower panel: a single bacterium. (i) Ratio of bacterial cells with flagella. For each strain, cells with flagella were counted (*n* = 100). (j) The average flagellar length of bacterial strains (*n* = 30). (k) *fliC* mRNA level in bacterial strains. The level of *fliC* mRNA was measured by qRT-PCR. Amplification of cDNA from tmRNA was used as an internal control. The experiment was repeated three times and the result of a representative experiment is shown. Standard deviations are provided; asterisk: significant difference, as tested by Student’s *t*-test (*P* ≤ 0.05).

Intriguingly, the RR RavR is upstream of RavS (HK) in regulation. The protein has an EAL domain and a GGDEF domain that associated with c-di-GMP turnover. Using ^32^P-labelled c-di-GMP produced by a tDGC cyclase (S5A Fig) [41], we confirmed that the EAL domain of RavR can form a homodimer *in vitro* and has phosphodiesterase activity to degrade c-di-GMP into pGpG, which depends on the E-A-L motif of the domain (Fig 4A, S5B Fig and S5C Fig). The GGDEF signature motif of RavR is degenerate as “GSDEM” and did not have diguanylate cyclase activity to synthesize c-di-GMP from GTP (S5B Fig), suggest that RavR does not encode cyclase activity. Phosphorylation of RavR by RavA did not affect its phosphodiesterase activity (S5D Fig and S5E Fig), consistent with the result that inactivation of two HK genes (*ravS* or *ravA*) did not remarkably affect the intracellular concentration of c-di-GMP (S5F Fig). Additionally, deletion of the coding sequences of the EAL domain or *ravR* gene increased the intracellular c-di-GMP to 185.9% and 150.0% of the level of the WT strain, respectively (Fig 4B). These results suggest RavR mainly acts as a phosphodiesterase.

We further genetically analysed the interaction between the phosphor-receiving domain (REC) and the EAL domain of RavR and their regulatory relationship in controlling *ravS*. Similar to the null mutant of *ravR* (ΔravR), point mutation of the conserved Asp^496^ residue within the REC domain of RavR decreased bacterial virulence and EPS production (Fig 4C–4E) but increased bacterial swimming (Fig 4F and 4G), flagellar cell ratio (Fig 4H and 4I), flagellar length (Fig 4J) and the transcriptional level of *fliC* (Fig 4K), which suggests that phosphorylation residue Asp^496^ of RavR is indispensable for regulation.

Surprisingly, inactivation of RavR phosphodiesterase activity by an in-frame deletion of the coding sequence of its EAL domain (ravR^ΔEAL^) caused phenotypic changes that were completely different from those of the *ravR* null mutant (ΔravR) or ravR^D496A^ point mutant. The virulence level and EPS production of ravR^ΔEAL^ were unaffected (Fig 4C–4E) rather than reduced. The swimming motility of the ravR^ΔEAL^ mutant was reduced significantly (Fig 4F and 4G) rather than showing a dramatic increase that was observed for ΔravR or the ravR^D496A^ mutant. TEM analysis revealed the absence of flagellum in the bacterial cells of the ravR^ΔEAL^ mutant; thus, its flagellar cell ratio was zero (Fig 4H–4J). Additionally, the *fliC* mRNA level of the ravR^ΔEAL^ mutant was reduced significantly (Fig 4K). We then constructed a double mutant of ravR^ΔEAL(D496A)^ to investigate the epistatic relationship between the EAL domain and Asp^496^. ravR^ΔEAL(D496A)^ phenocopied the ravR^D496A^ mutant displaying a decrease in virulence and EPS production but increases in swimming capability, flagellar length, flagellar cell ratio and *fliC* transcription level (Fig 4A–4K). This genetic evidence suggests that RavR has two opposite functions in regulating bacterial swimming. Full-length RavR negatively controls swimming and flagellar development in an Asp^496^-dependent manner, whereas its EAL domain positively controls swimming and flagellar development. Additionally, epistatic analysis revealed that the phosphorylation of Asp^496^ potentially downstream of the PDE activity of EAL domain in regulating bacterial swimming. The biochemical relationship will be dissected in the following experiments.

Based on the above results, the genetic code of the conserved His^500^ residue of the downstream HK gene *ravS* was mutated on the backgrounds of ravR^D496A^ and ravR^ΔEAL(D496A)^ to construct triple mutants. As predicted, the pattern of phenotypic changes in these three triple mutants was suppressed by the mutation and similar to that of ΔravS or ravS^H500A^ (Fig 4A–4K).

### Both RavA and RavS phosphorylate RavR

The phosphorylation process and regulation of RavA-RavR-RavS have not been investigated previously. *In vitro* phosphorylation assays using autoradiography and Phos-tag gel analysis revealed that full-length RavA autophosphorylates its conserved RavA^His164^ residue because the RavA^H564A^ substitution completely inactivated RavA autophosphorylation (S6A Fig, S6B Fig and S6D Fig). The addition of RavR or RavR^ΔEAL^ into the reaction mixture remarkably decreased the RavA phosphorylation level (RavA~P), although no recognisable signal of phosphorylated RavR (RavR~P or RavR^ΔEAL^~P) was detected in autoradiography (S6A Fig, S6B Fig and S6D Fig). Recombinant RavR with a conserved Asp^496^ substitution did not cause a decrease in the RavA~P level (S6A Fig, S6B Fig and S6D Fig), which suggests that RavA transfers the phosphoryl group onto Asp^496^ of RavR and that RavR~P is highly unstable. Unlike isotope-labelling method, Phos-tag gel analysis successfully detected a RavR~P band in the RavA-RavR phosphotransfer reaction under an extremely high ATP concentration (2 mM, S6D Fig, lane 5), providing an evidence to demonstrate that RavA phosphorylates RavR.

**Fig 5:**
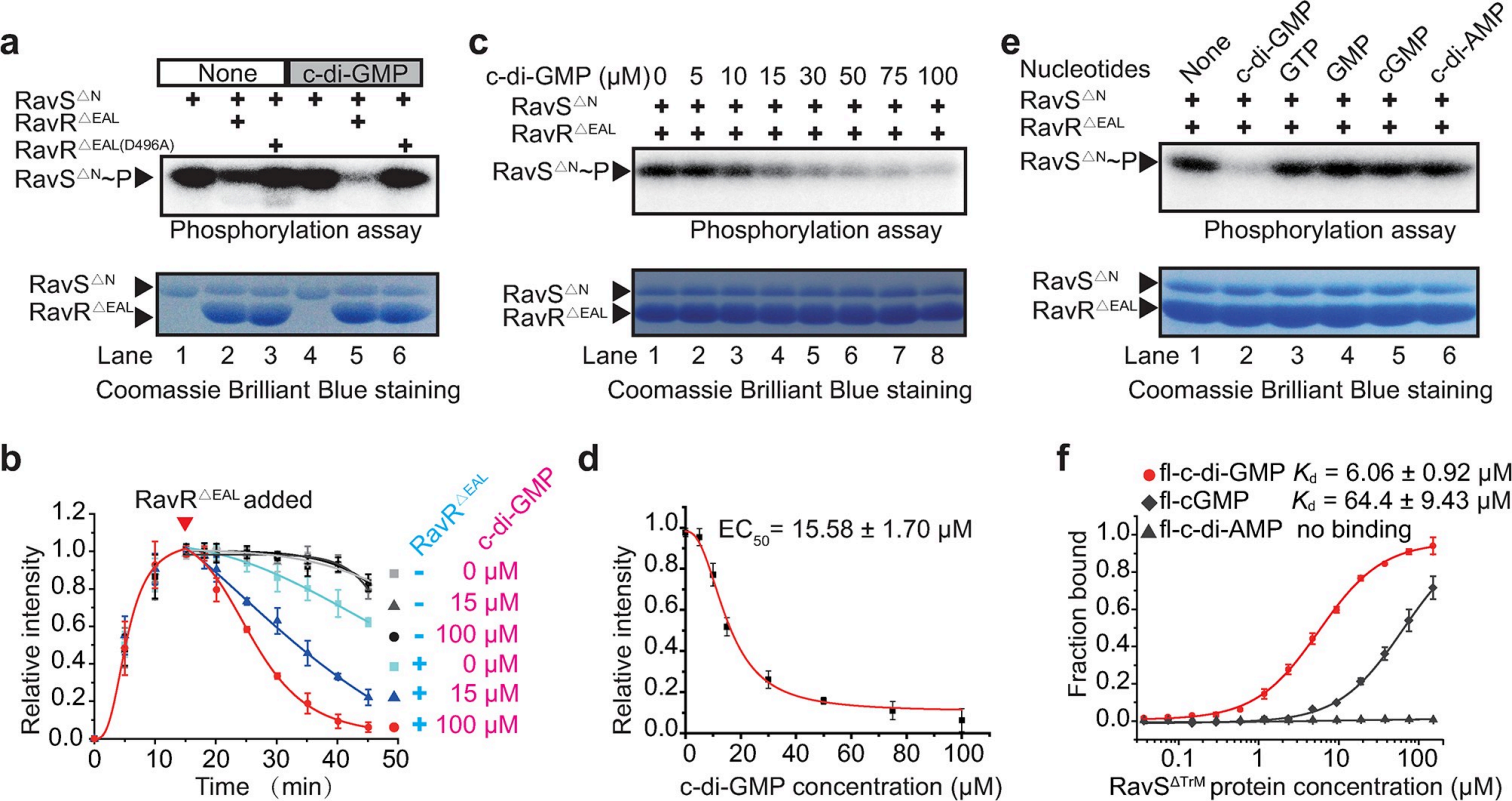
c-di-GMP specifically and significantly enhances RavS-RavR phosphotransfer. (a) c-di-GMP enhances RavS-RavR phosphotransfer *in vitro*. A total of 5 μM RavS^ΔN^ was autophosphorylated for 15 min by the addition of 10 μCi [γ-^32^P]ATP. A total of 15 μM RavR^ΔEAL^ or RavR^ΔEAL(D496A)^ was added and incubated for 30 min at 25°C. A total of 100 μM c-di-GMP was added if necessary. The reactions were terminated with 5× SDS loading buffer before autoradiography. The experiment was repeated three times. (b) Time course analyses of the impact of c-di-GMP on the velocity of RavS-RavR phosphotransfer. The autophosphorylation and phosphotransfer reactions were set as mentioned in (a). Various concentrations of c-di-GMP were added to the reactions and isotopic signals (band intensities) of RavS~P were measured after autoradiography. (c–d) c-di-GMP triggered an increase in RavS-RavR phosphotransfer in a dose-dependent manner. (c) Various concentrations of c-di-GMP (0, 5, 10, 15, 30, 50, 75 and 100 μM) were added to the RavS-RavR phosphotransfer reactions. (d) The phosphorylated level of RavS^ΔN^~P was quantified by ImageJ and the EC_50_ value was estimated by Origin 8.5 software. In (b) and (d), the intensity of each data point is the average from three experimental repeats. (e) c-di-GMP triggered increase in RavS-RavR phosphotransfer is highly specific. Other nucleotides, including GTP, GMP, cGMP and c-di-AMP, did not affect the velocity of the RavS-RavR phosphotransfer. (f) RavS specifically interacts with fl-c-di-GMP. The interaction between RavS^ΔTrM^ and various nucleotides, including fl-c-di-GMP, fl-cGMP and fl-c-di-AMP, were measured by microscale thermophoresis (MST). A total of 20 nM fluorescein-labelled nucleotides and increasing concentrations of proteins were incubated and measured by MST. The dissociation constant (*K*_d_) was quantified to estimate the binding affinity. Each experiment was repeated three times.

For RavS, repeated efforts failed to detect any signal representing the autophosphorylated RavS-containing cytosolic region (RavS^ΔTrM^, S6C Fig). However, a truncated RavS that lacks the N-terminal transmembrane helix and the two PAS domains (RavS^ΔN^) exhibited strong autokinase activity in the presence of ATP (Fig 5A, lane 1 and S6C Fig). The addition of RavR^ΔEAL^ to the reaction mixture slightly decreased the level of RavS~P after 30 min of co-incubation (Fig 5A, lane 2, S6E Fig, lane 3). No signals representing RavR~P were observed by the isotope-labelling and Phos-tag gel analysis (Fig 5A, lane 3, S6E Fig and S6F Fig) and substitution of the conserved Asp^496^ of RavR (RavR^ΔEAL(D496A)^) eliminated its activity to decrease the RavS~P level. Together, these biochemical analyses demonstrated that RavA and RavS are *bona fide* HKs. RavA can phosphorylate RavR at residue Asp^496^, and RavR~P is highly unstable.

### c-di-GMP specifically and significantly enhances RavS-RavR phosphotransfer

Since *ravS* is downstream of *ravR* during the regulation of bacterial swimming and RavR is a functional phosphodiesterase that degrades c-di-GMP, we reasoned that c-di-GMP modulates the activity of RavS in controlling bacterial swimming. We analysed the impact of c-di-GMP on the enzymatic activities of RavS to test this hypothesis.

In the absence or presence of c-di-GMP (Fig 5A, lane 1 and lane 4), the autokinase activity of RavS^ΔN^ was unchanged, which suggests that c-di-GMP does not affect RavS^ΔN^ autophosphorylation. However, when RavR^ΔEAL^ was added to the RavS-RavR phosphotransfer reaction, the presence of c-di-GMP in the mixture remarkably increased RavS-RavR phosphotransfer because the RavS^ΔN^~P level immediately decreased when compared with the level in the absence of c-di-GMP (Fig 5A, lane 5 vs. lane 2, and Fig 5B). The time (T_50_) that signal intensity of the RavS~P decreased to the half of the initiation stage (before adding RavR^ΔEAL^) was estimated: without c-di-GMP, the T_50_ value of RavS~P is 38.2 min. When 15 μM c-di-GMP is in presence, the T_50_ value of RavS~P remarkably decreased to 16.9 min. Since some HKs or RRs have phosphatase activity to dephosphorylate HKs, it is possible that c-di-GMP positively regulates RavS or RavR phosphatase activity to decrease the RavS~P level. However, addition of c-di-GMP alone did not affect RavS^ΔN^~P levels (Fig 5A, lane 4 vs. lane 1, and Fig 5B) and the addition of an inactive RavR^ΔEAL(D496A)^ did not have any impact on RavS phosphorylation levels, regardless of the presence or absence of c-di-GMP (Fig 5A, lanes 3 and 6). These results indicate that c-di-GMP enhanced significantly RavS phosphotransferase activity towards RavR. This c-di-GMP-triggered kinetic preference effectively decreases the RavS~P level.

Along with the elevation of c-di-GMP concentrations, the RavS-RavR phosphotransfer, which was measured by the intensity of the RavS~P signal, gradually increased. This suggests that the impact of c-di-GMP on RavS autokinase activity is dose-dependent (Fig 5C). The estimated value of the median effect concentration (EC_50_) of c-di-GMP was approximately 15.58 μM (Fig 5D). Furthermore, the regulation of c-di-GMP on the RavS-RavR phosphotransfer to decrease the RavS~P level is highly specific because that other nucleotides and derivatives, including GTP, GMP, cGMP and c-di-AMP, did not have any impact on RavS phosphotransferase activity (Fig 5E).

### Arg^656^ within the CA region of RavS is essential for c-di-GMP binding

**Fig 6:**
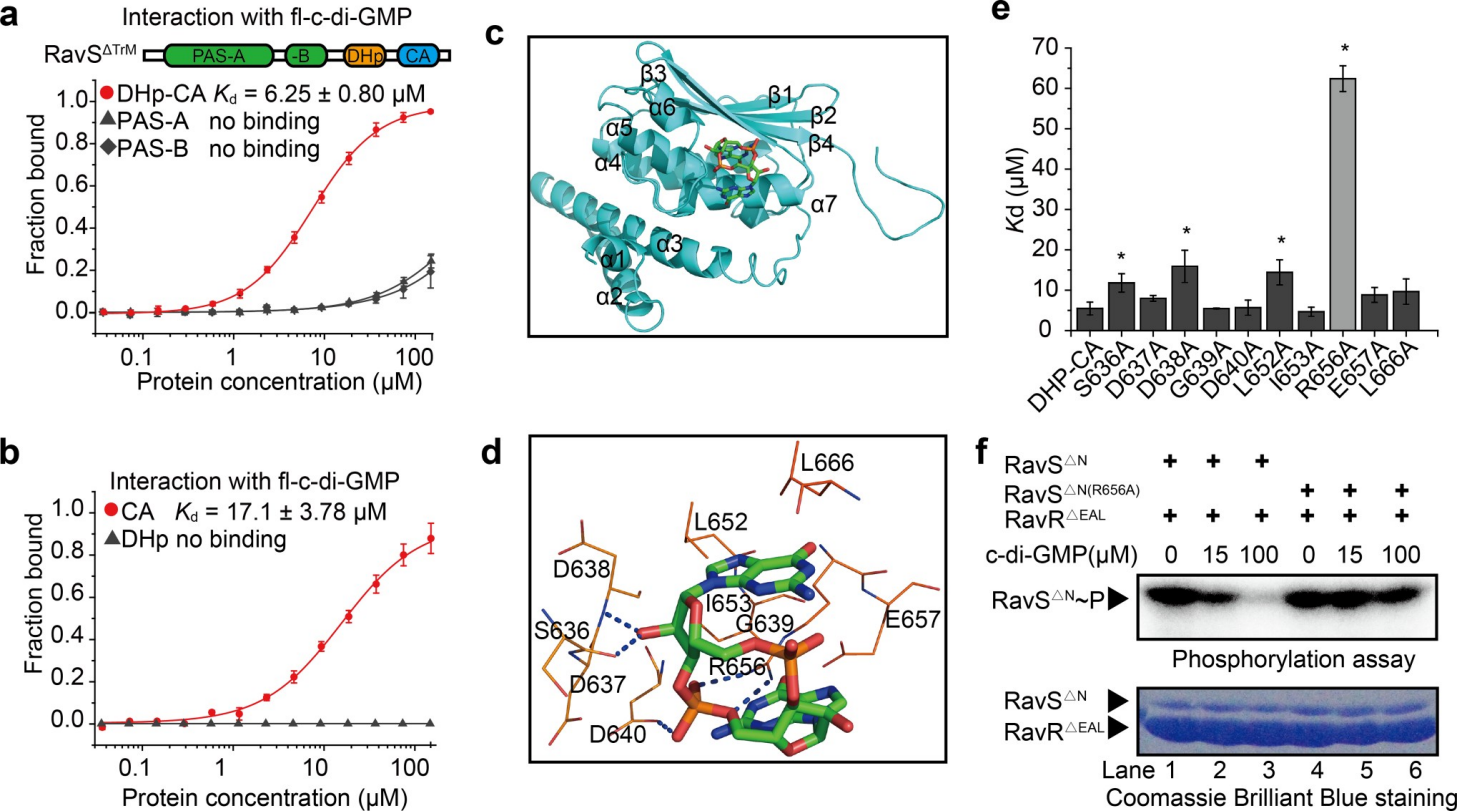
Arg^656^ in the CA domain of RavS is a key residue for c-di-GMP binding. **(**a, b) c-di-GMP binds to the DHp-CA domain of RavS. PAS-A, PAS-B and DHp-CA in (a), and DHp and CA domains of RavS in (b), were purified and the interaction with c-di-GMP was measured by MST. Each assay was repeated three times. Standard deviations are shown. (c) Molecular docking model of the interaction between c-di-GMP and DHp-CA of RavS. c-di-GMP is shown as a ball-and-stick model and DHp-CA is shown in ribbon representation. (d) Schematic view of the predicted docking site between c-di-GMP and DHp-CA. Potential hydrogen bonds are indicated as blue dashed lines. Autodock software was used to predict the interaction. (e) Effect of amino acid substitutions on RavS–c-di-GMP binding. Ten recombinant proteins of the DHp-CA region, each containing a substitution of a putative essential residue involved in c-di-GMP binding, were used in the MST assay to quantify the *K*_d_ value of RavS–c-di-GMP binding. fl-c-di-GMP was used in the MST assay. Each experiment was repeated three times. (f) Substitution of Arg^656^ of RavS resulted in the loss of RavS-RavR phosphotransfer upon stimulation by c-di-GMP. A total of 5 μM RavS^ΔN^ or RavS^ΔN(R656A)^ was phosphorylated by 10 μCi [γ-^32^P]ATP for 15 min in the absence or presence of 15 or 100 μM c-di-GMP, and then 15 μM RavR^ΔEAL^ was added and incubated for 30 min at 25°C. The reactions were terminated with 5× SDS loading buffer and the products were separated by 12% SDS-PAGE, exposed to a phosphor screen and analysed by Typhoon FLA7000. The experiment was repeated three times.

Next, microscale thermophoresis (MST) was used to determine whether c-di-GMP binds directly to RavS. 2′-Fluo-AHC-c-di-GMP (fl-c-di-GMP) was used in the assay since the analogue contains a carboxyfluorescein that has excitation and emission wavelengths of 497 nm and 520 nm, respectively, which can be detected directly by a MST instrument [42]. As shown in Fig 5F, fl-c-di-GMP directly bound a truncated, soluble RavS protein lacking the transmembrane region (RavS^Δ^). The dissociation constant was 6.06 ± 0.92 μM, which is in the range of physiological concentrations of c-di-GMP in bacterial cells [22]. As controls, 2′-Fluo-AHC-cGMP (fl-cGMP) or 2′-Fluo-AHC-c-di-AMP (fl-c-di-AMP) bound RavS^ΔTrM^ with weaker affinity (fl-cGMP, 64.4 ± 9.43 μM) or completely failed to bind the protein (fl-c-di-AMP, Fig 5F). Furthermore, to determine the c-di-GMP binding region on RavS, we expressed and purified various RavS fragments (S7A Fig). MST assays revealed that fl-c-di-GMP bound RavS with the DHp-CA or CA region, whereas this second messenger did not bind the PAS-A, PAS-B and DHp regions of RavS (Fig 6A and 6B). Of note, a lower binding affinity of c-di-GMP with the CA domain than the DHp-CA domain was observed, which suggests that the DHp domain indirectly affects the interaction (Fig 6B). MST analysis also revealed that fl-c-di-GMP did not bind to RavR^ΔEAL^, RavA and an unrelated HK VgrS since the *K*_d_ values were greater than 80 μM (S7B Fig), which did not have physiological significance. Therefore, these results revealed that c-di-GMP specifically binds to RavS and the CA domain of RavS is the possible interacting region of c-di-GMP.

Similar to most HKs, the three-dimensional (3D) structure of RavS has not been elucidated. As an alternative, molecular docking was used to predict the structural interaction of the RavS–c-di-GMP complex. We predicted the DHp-CA (residues E464–R697) structure of RavS by homology modelling with the crystal structures of other HKs, such as CpxA-HDC (PDB ID: 4BIU), DivL (4EW8) and VraS (4GT8), as templates. As shown in Fig 6C, the putative structure of DHp-CA is composed of seven α-helices, four β-strands and random crimps. The active centre includes the helices of α3, α4, α5 and α6 and strands β3 and β4, and is highly similar to other DHp-CA domains of HKs. The 3D structure of c-di-GMP was extracted from the crystal structure of the c-di-GMP-VCA0042 complex (PDB ID: 2RDE) [43]. Autodock 4.0 software was used for molecular docking. The results showed that helices α4 and α7 and strands β3 and β4 of the RavS DHp-CA domain formed a c-di-GMP binding pocket and 10 residues in the vicinity of the pocket potentially interact with c-di-GMP (Fig 6C and 6D). Among these residues, Ser^636^, Asp^638^, Asp^640^, Ile^653^, Glu^657^ and Leu^666^ putatively interact with c-di-GMP via hydrophobic interactions and van der Waals forces, whereas Asp^637^, Gly^639^, Leu^652^ and Arg^656^ form hydrogen bonds with c-di-GMP (Fig 6D). In contrast to the other residues, the amino hydrogens of Arg^656^ and the phosphate group oxygens of c-di-GMP formed two hydrogen bonds with bond lengths of 2.3 Å and 2.5 Å, respectively (Fig 6D). These results suggest that c-di-GMP potentially interacts with the CA region of RavS and that Arg^656^ is a key residue in the interaction.

To experimentally verify the above prediction, we expressed and purified 10 recombinant DHp-CA proteins in which each of the predicted c-di-GMP-interacting amino acids were separately substituted to Ala. MST assays revealed that the recombinant proteins DHpCA^D637A^, DHp-CA^G639A^, DHp-CA^D640A^, DHp-CA^I653A^, DHp-CA^E657A^ and DHp-CA^L666A^ exhibited no significant change in c-di-GMP binding affinity. In contrast, DHp-CA^S636A^, DHp-CA^D638A^ and DHp-CA^L652A^ exhibited 1–2-fold increases in *K*_d_ values when compared with that of the positive control (DHp-CA protein), which suggests that these substitutions disrupt the protein-nucleotide interaction. Notably, the *K*_d_ value of the c-di-GMP–DHp-CA^R656A^ interaction was 62.4 ± 3.19 μM, which showed a 10-fold decrease in binding affinity (Fig 6E). Consistent with this result, an *in vitro* phosphotransfer assay revealed that c-di-GMP failed to stimulate the phosphotransferase activity of RavS^ΔN(R656A)^~P towards RavR^ΔEAL^ and the RavS^ΔN(R656A)^~P level did not change noticeably or decreased slightly in the presence of c-di-GMP (15 or 100 μM, Fig 6F). Thermal shift assay revealed that the DHp-CA^R656A^ is as stable as DHp-CA protein (S8A Fig), and RavS^ΔN^ and RavS^ΔN(R656A)^ have similar autokinase activity (S8B Fig), suggesting that the Arg^656^ substitution did not remarkably affect the structure of the recombinant protein. Collectively, these results demonstrate that Arg^656^ of RavS is a key residue in c-di-GMP binding. Substitution of Arg^656^ leads to dissociation between c-di-GMP and RavS such that the RavS-RavR phosphotransfer is not accelerated. Under these circumstances, the majority of RavS remains phosphorylated.

### RavS^R656A^ substitution causes phenotypic changes that mimic constitutively activated RavS

**Fig 7:**
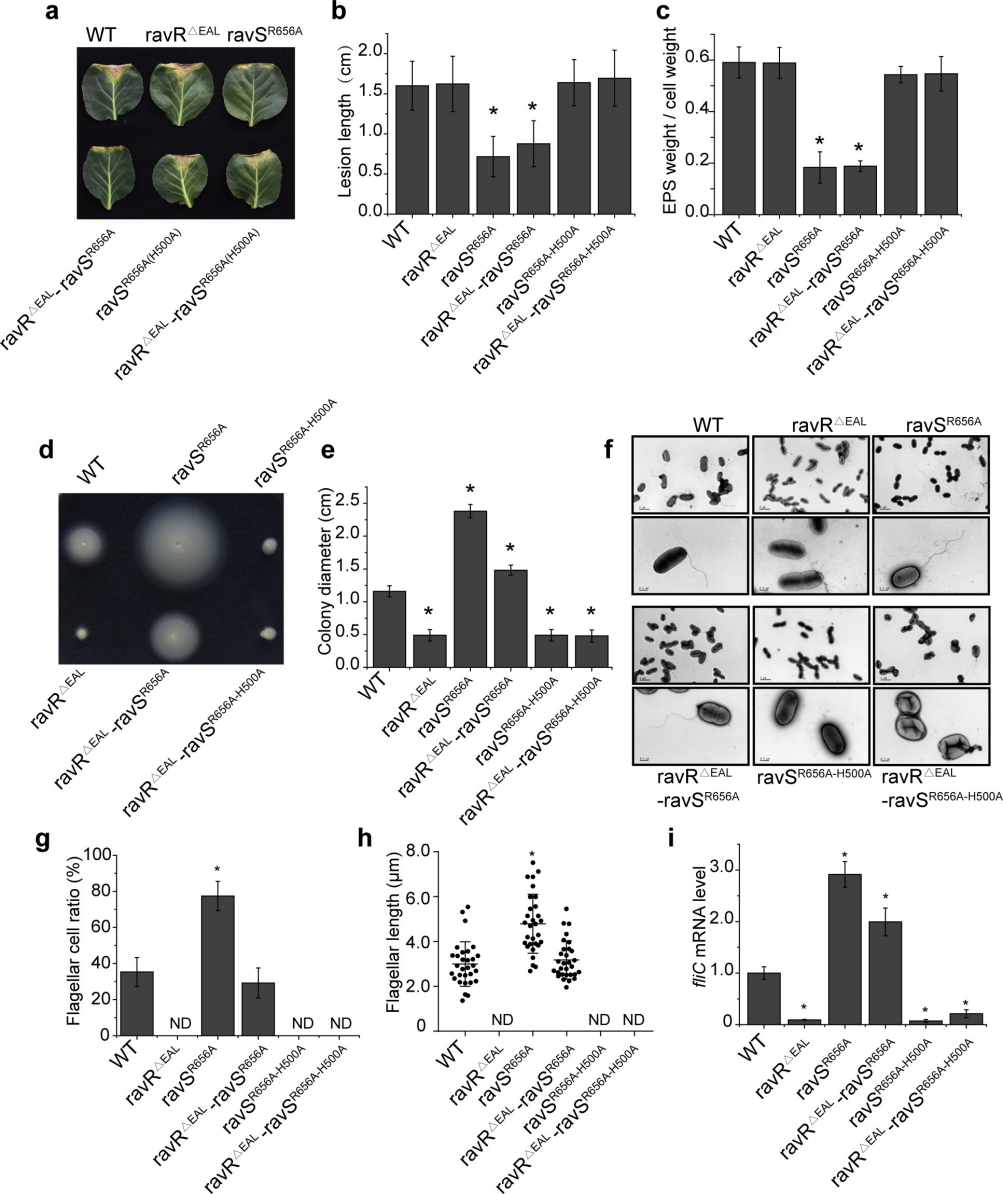
ravS^R656A^ suppresses a phenotypic deficiency caused by ravR^ΔEAL^ in a RavS His^500^-dependent manner. (a, b) Bacterial virulence. Bacterial strains were inoculated onto plant leaves of *Brassica oleracea* cv. Zhonggan 11. The lesion length was recorded 10 d after inoculation (*n* = 30). (c) Production of extracellular polysaccharides (EPS). EPS quantification was calculated as the dry weight of EPS vs. the dry weight of bacterial cells (*n* = 3). (d, e) Bacterial swimming motility. (d) Bacterial strains were inoculated in NYG plates containing 0.15% agar and grown at 28°C for 28 h. (e) Average diameters of the migration zones were measured (*n* = 10). (f) Morphology of bacterial flagella. Bacterial flagella were observed by TEM after negative staining. A representative image of each strain is shown. Upper panel: bacterial population profile; lower panel: single bacterium. (g) Ratio of bacterial cells with flagella. For each strain, cells with flagella were counted (*n* = 100). (h) The average flagellar length of bacterial strains (*n* = 30). (i) *fliC* mRNA levels in bacterial strains. The amount of *fliC* mRNA was measured by qRT-PCR. Amplification of cDNA from tmRNA was used as an internal control. The experiment was repeated three times and the result of a representative is shown. In the figure, standard deviations are shown; asterisk: significant difference, as tested by Student’s *t*-test (*P* ≤ 0.05). Three biological replicates were performed.

Based on the above results, we constructed a ravS^R656A^ point mutant to determine whether the dissociation of c-di-GMP from RavS affects bacterial phenotypes. Western blotting revealed that RavS is stable in this mutant (S8C Fig). As shown in Fig 7A and 7B, the virulence of the ravS^R656A^ mutant was attenuated substantially and the production of EPS by this mutant had also decreased significantly (Fig 7C). The swimming zone diameter of the ravS^R656A^ mutant was 2.38 ± 0.10 cm, which was much larger than that of the WT strain (1.16 ± 0.08 cm) (Fig 7D and 7E). Therefore, we measured the parameters of bacterial flagella and found that the ravS^R656A^ mutant had longer flagella (4.79 ± 1.31 μm, Fig 7F and 7H). The ravS^R656A^ mutant flagellar cell ratio was two-times the level of the WT strain (Fig 7G) and the *fliC* mRNA level had increased by 292% when compared with that of the WT strain (Fig 7I). Therefore, the pattern of phenotypic changes of the ravS^R656A^ point mutant is similar to that of the *ravS* overexpression strain (ΔravS-ravS; S9A–S6F Figs) but is quite different from that of the *ravS* deletion or ravS^H500A^ mutant whose virulence and EPS production were unaffected, whereas the tested parameters of swimming motility were reduced significantly (Fig 3).

Thus, these results suggest that the ravS^R656A^ substitution, which eliminated the binding capability of c-di-GMP, mimics the constitutive phosphorylated state of RavS. Subsequently, the conserved His^500^ residue was mutated on the genetic background of ravS^R656A^. Compared with the ravS^R656A^ mutant, the double mutant ravS^R656A-H500A^ exhibited restored virulence and EPS production levels (Fig 7A–7C). The swimming motility (Fig 7D and 7E), flagellar cell ratio (Fig 7F and 7G), flagellar length (Fig 7H) and *fliC* mRNA levels (Fig 7I) of the double mutant were all significantly reduced or undetectable. Therefore, point mutation of His^500^ completely suppressed the mutational effects caused by the Arg^656^ point mutation. Together with the biochemical results demonstrating that c-di-GMP did not bind ravS^R656A^ and that the phosphorylation level of RavS^R656A^ is insensitive to c-di-GMP inhibition, these findings suggest that c-di-GMP associates with the Arg^656^ residue to negatively regulate the RavS phosphotransferase activity towards RavR.

### RavR regulates RavS~P levels through a c-di-GMP-enhanced phosphate sink

The aforementioned genetic and biochemical results prompted the following hypothesis: a high level of RavS~P positively regulates bacterial swimming but is detrimental to virulence; however, dephosphorylation of RavS reduces the expression of *fliC* and is negative in modulating bacterial swimming. Since c-di-GMP binds to RavS to inhibit the phosphotransferase activity of RavS towards RavR, RavR is likely to be a special phosphate sink that controls the RavS~P level through opposite mechanisms: 1) the REC domain of RavR receives the phosphoryl group from RavS~P, which decreases the RavS~P level and then negatively regulates bacterial swimming. Additionally, c-di-GMP inhibits bacterial swimming by further enhancing the RavS-RavR phosphotransfer; and 2) the EAL domain of RavR degrades c-di-GMP and then inhibits the c-di-GMP-triggered RavS-RavR phosphotransfer. This process prefers to maintain the RavS~P level and positively regulates bacterial swimming. This hypothesis is true when constitutive dissociation by genetic manipulation of the binding event between c-di-GMP and RavS results in a high RavS~P level. As a consequence, this process will positively modulate bacterial swimming independent of the c-di-GMP concentration in cells.

To test this hypothesis, we point mutated Arg^656^ of *ravS* to Ala to construct a double mutant on the ravR^ΔEAL^ genetic background (ravR^ΔEAL^ravS^R656A^). The RavR phosphodiesterase activity of this mutant was genetically inactivated and recombinant RavS did not bind c-di-GMP because Arg^656^ is essential for the protein-[c-di-GMP] interaction. As shown in Fig 7, the point mutation of *ravS*^R656A^ effectively suppressed the phenotypic effects (i.e., virulence and EPS production) caused by the EAL domain deletion (Fig 7A–7C) and bacterial swimming was restored completely (1.48 ± 0.08 cm; Fig 7D and 7E). The number of flagella and flagellar cell ratio were increased significantly when compared with the level of the WT strain (Fig 7F–7H). The *fliC* mRNA level of the ravR^ΔEAL^ravS^R656A^ mutant was also significantly increased from 9.4% (ravR^ΔEAL^) to 199% of the level of the WT strain (Fig 7I). Similarly, overexpression of *ravS* on the ravR^ΔEAL^ ΔravS background (ravR^ΔEAL^ΔravS-OE-ravS; S9A Fig), which mimics constitutive phosphorylation of RavS, also effectively suppressed the mutational effects of the EAL domain deletion on bacterial swimming (S6 Fig).

Furthermore, the suppression of the ravS^R656A^ mutation and ravR^ΔEAL^ deletion completely depends on the conserved His^500^ site of RavS. The triple mutant of ravR^ΔEAL^ravS^R656A(H500A)^ exhibited performance similar to that of ravR^ΔEAL^ in all of the tested parameters associated with swimming or flagella development (Fig 7A–7I). Collectively, these genetic analyses support the hypothesis regarding the regulation of RavR in controlling the RavS~P level. Asp^469^ within the REC domain of RavR receives the phosphoryl group from RavS to reduce the RavS~P level, whereas the EAL domain of RavR degrades c-di-GMP, which binds to RavS and promotes the specificity between RavS-RavR by broadening the phosphotransfer flux. Dissociation of RavS–c-di-GMP leads to enhanced bacterial swimming, which depends on the high phosphorylation level of RavS.

## Discussion

**Fig 8:**
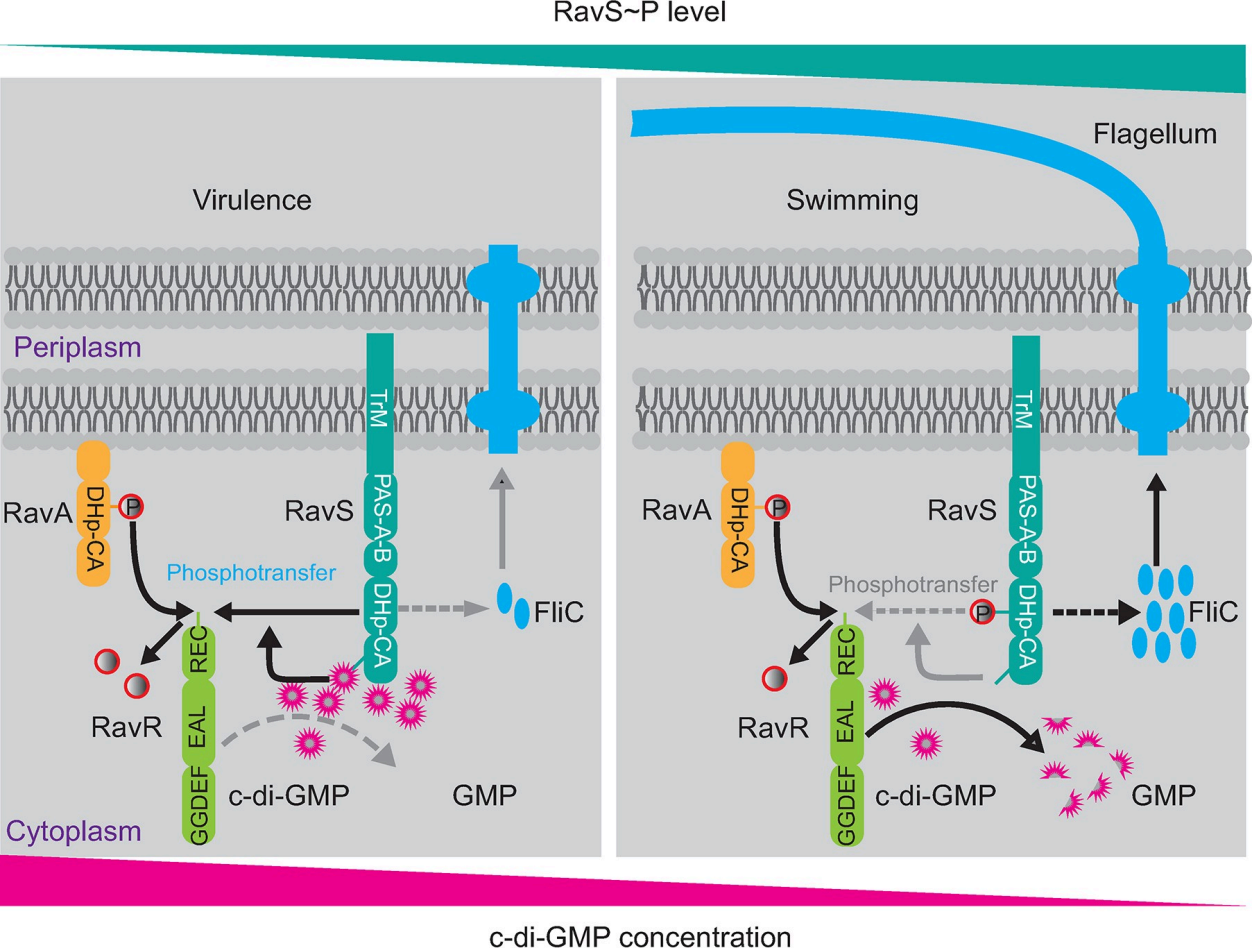
c-di-GMP–RavS binding promotes the specificity of the RavS–RavR phosphotransfer to control the bacterial lifestyle transition between swimming and virulence. Left panel: During infection, RavA–RavR phosphotransfer is necessary to control bacterial virulence, whereas RavS is maintained at a low phosphorylation level. c-di-GMP binds to RavS to enhance the phosphotransfer to the REC domain of RavR, which acts as a phosphate sink. Right panel: During bacterial swimming, the level of phosphorylated RavS is high. The EAL domain of RavR is activated and degrades the c-di-GMP binding to RavS. This process decreases RavS-RavR phosphotransfer and promotes an increase in the RavS~P level to regulate flagella development. TrM: transmembrane helix. DHp: dimerization and histidine phosphotransfer domain. CA: catalytic and ATP binding domain. REC: receiver domain. P in the red circle represents the phosphoryl group. Grey dashed arrows or terms indicate that the biochemical processes were inactivated.

Kinetic preferences between HKs and RRs define the specificity of TCS regulation [7, 44], which is quantified by the velocity of a HK to phosphorylate a RR [6]. In the present study, genetic, biophysical and biochemical data revealed that c-di-GMP substantially enhanced the specificity between RavS and RavR of *X*. *campestris* pv. *campestris*. RavS physically interacts with c-di-GMP via its CA region. This interaction remarkably accelerated phosphoryl transfer from RavS~P towards RavR, resulting in efficient dephosphorylation of RavS~P. Intriguingly, the EAL domain-containing RavR acts as a phosphate sink of RavS~P. Stimulated by c-di-GMP, the REC domain of RavR receives a phosphoryl group from RavS to decrease the RavS~P level, which negatively modulates bacterial swimming. However, the EAL domain of RavR degrades c-di-GMP. Degradation of c-di-GMP revokes the accelerating effect of c-di-GMP on RavS-RavR phosphotransfer, leading to a high level of RavS~P, which positively regulates bacterial swimming (Fig 8). Therefore, these results suggest that RavS is a pivotal node for integrating the regulatory pathways of c-di-GMP and TCS, and RavR is a bifunctional regulator that finely controls the phosphorylation state of RavS (Fig 8). The crosstalk between c-di-GMP and RavS–RavR phosphoryl transfer regulates a bacterial lifestyle transition from virulence to freely swimming.

c-di-GMP plays a pleiotropic role in controlling the physiological processes of bacterial cells. This cyclic dinucleotide is a versatile, flexible molecule and the regulatory function of c-di-GMP is usually exerted by binding to a diverse array of effectors to elicit allosteric changes [14, 18, 45]. However, the intracellular concentration of c-di-GMP is complex and changed dynamically [46]. The local, subcellular c-di-GMP concentrations are critical and have profound effects on physiological processes [14]. Currently, the reported *K*_d_ values between c-di-GMP and proteins range from 0.1 μM (PizD) to 14.5 μM (STING) [22]. Our MST assay revealed that c-di-GMP physically binds to RavS *in vitro* with a *K*_d_ of 6.06 μM (Fig 5F), which is similar to the previously identified effector CLP of *Xanthomonas campestris* pv. *campestris* [47], suggesting that RavS is a *bona fide* c-di-GMP effector. The binding of c-di-GMP to RavS substantially increased its phosphotransferase activity towards RavR but did not affect RavS autokinase and phosphatase activities (Fig 5A and 5B). In TCS regulation, phosphotransfer from HK to RR is generally controlled by their specificity, which is defined by key ‘specificity residues’ in these proteins [6, 44]. However, our results showed that c-di-GMP did not bind the RR RavR (S7B Fig), thus excluding the possibility that c-di-GMP increases RavR phosphatase activity towards RavS~P. Since the DHp and CA domains of HK are associated, we reasoned that the binding of c-di-GMP to the CA domain confers a conformational switch in the DHp domain, which leads to more efficient phosphoryl transfer from the DHp domain to the REC domain of RavR. Details on c-di-GMP-mediated enhancement of phosphoryl transfer require further structural analysis.

According to previous studies, c-di-GMP binds to diverse regions of proteins, such as the degenerate GGDEF and EAL domains, PilZ domain and the interdomain linker [22]. In the absence of the RavS structure, we employed a molecular docking method together with mutational analysis to define residues essential to interaction with c-di-GMP (Fig 6). The analysis suggests that Arg^656^ within the helix α7 region of the CA domain potentially forms two hydrogen bonds with c-di-GMP (Fig 6C and 6D). Substitution of this residue eliminated RavS–c-di-GMP binding *in vitro* (Fig 6E). Additionally, point mutant ravS^R656A^ exhibited enhanced bacterial swimming, elongation of flagella, a higher ratio of flagellar cells and increased *fliC* transcription levels (Fig 7). Similar to the overexpression of *ravS* (S9 Fig), the ravS^R656A^ mutation suppressed completely the mutational effects of the ravR^ΔEAL^ deletion that decreased swimming motility (Fig 7). Therefore, Arg^656^ within the CA region is an essential residue for binding c-di-GMP. This finding is in accordance with the preference of Arg or Asp/Glu residues to form hydrogen bonds with the phosphate group of c-di-GMP [22]. Compared with CckA of *C*. *crescentus*, which also binds to c-di-GMP, both HKs contain two PAS domains and a DHp-CA core; however, RavS lacks the C-terminal REC domain and is not a hybrid-type HK. When interacting with c-di-GMP, the autokinase activity of CckA was inhibited but its phosphatase activity was increased [27–29]. However, c-di-GMP binding did not affect the RavS autophosphorylation. It increases the phosphoryl flux from RavS towards RavR. The structural basis of this difference is unclear.

Intriguingly, RavR plays a special role in regulation. Previous studies on c-di-GMP turnover found a general trend in which an elevated cellular concentration of c-di-GMP leads to a reduction in motility behaviours [14, 18, 48]. Therefore, inactivation of EAL domain-containing proteins usually increases c-di-GMP concentrations and inhibits bacterial swimming. In this study, deletion of the *ravR* gene increased the cellular concentration of c-di-GMP (Fig 4B) but this increase promoted bacterial swimming substantially rather than reducing this activity (Fig 1G). Further investigation revealed that deletion of the EAL domain of RavR caused completely opposite phenotypic changes as the swimming zone was significantly reduced (Fig 4F). This phenotype led us to examine the function of the EAL and REC domains of RavR. We found that RavR acts as a phosphate sink of RavS. Since the point mutation of ravR^D496A^ restored the reduced swimming motility caused by EAL deletion (Fig 4F–4K), the Asp^496^-dependent phosphoryl-receiving activity of RavR functions to regulate RavS~P levels by c-di-GMP-triggered RavS-RavR phosphotransfer, rather by directly modulating the PDE activity of the EAL domain, as revealed by S5D–S5F Fig. Based on these findings, the EAL domain degrades cellular c-di-GMP to release the negative regulation of c-di-GMP at the RavS~P level by promoting RavS-RavR phosphotransfer. Therefore, RavR is a bidirectional regulator that controls the RavS~P level through degrading c-di-GMP and accepting phosphoryl group from RavS.

As genetic and biochemical analyses revealed, both HKs RavA and RavS phosphorylate RavR, and all three proteins control the virulence and swimming of *X*. *campestris* pv. *campestris* (Figs 1 and 5). Among these proteins, RavA and RavR compose a typical TCS that regulates bacterial virulence. As a previous study revealed, *ravR* is downstream of *ravA* in regulation [37, 38]. In *X*. *oryzae* pv. *oryzae*, a close relative of *X*. *campestris* pv. *campestris*, *ravA*-*ravR* orthologues (*pdeK*-*pdeR*) have the same relationship in modulating bacterial blight disease against rice (*Orzya sativa*) [49]. As mentioned above, *ravR* is upstream of *ravS* in regulating bacterial swimming because RavR acts as a phosphate sink to dephosphorylate RavS (Fig 3). Our results further suggest that the regulation of bacterial virulence by RavA–RavR also depends on RavS because the *ravS* mutation suppressed the deficiencies of *ravA* and *ravR* mutants in virulence as well as in swimming (Figs 3 and 7). Since RavS and RavA are HKs that detect environmental and intracellular stimuli, it is important to know what signals were monitored by the two HKs. Because RavA lacks a recognizable signal input domain, it is challenging to predict the stimulus detected by RavA. However, RavS contains two intracellular PAS domains. PAS domain was found to sense low oxygen, redox, light or binds to small chemicals [50, 51]. A previous study also demonstrated that hypoxia sensing by RavS is involved in regulating virulence of *X*. *campestris* [37]. In our study, we found that the truncated RavS without the N-terminal transmembrane and PAS domains had a higher level of autokinase activity, indicating that the PAS domains autoinhibit the phosphorylation of RavS. If this hypothesis is true, the signals detected by PAS domains, such as low oxygen and still to-be-identified stimuli, will regulate RavS phosphorylation and play important roles in controlling virulence and motility of *X*. *campestris*.

*In vitro* phosphotransfer assays revealed that upon phosphorylation by RavA or RavS, there is no signal representing the observed RavR~P, which suggests that RavR~P is highly unstable (Fig 5A and S6 Fig). This biochemical feature of RavR may have an essential role in regulation because regardless of whether the phosphoryl groups are obtained from RavS or RavA or even if the transfer speed is enhanced by c-di-GMP, RavR can be promptly uncharged and avoid rapid saturation in phosphorylation. The unstable nature of RavR~P make the protein to be uncharged by dephosphorylation very quickly [52, 53]. Furthermore, considering that dissociation of the interaction between RavS and c-di-GMP either by substitution of Arg^656^ or by overexpression of *ravS* led to virulence attenuation, we speculate that RavR~P dephosphorylation has an important role in regulating the lifestyle transition between virulence and swimming motility. Future investigations are needed to dissect the molecular mechanism controlling the half-life of RavR~P in the context of RavA-RavR-RavS regulation.

## Materials and methods

### Bacterial strains, plasmids and culture conditions

All bacterial strains and recombinant vectors used in this work are listed in S1 Table. The WT strain of *X*. *campestris* pv. *campestris* ATCC 33913 and recombinant strains were grown at 28°C in rich NYG medium (5 g L^−1^ tryptone, 3 g L^−1^ yeast extract and 20 g L^−1^ glycerol, pH 7.0). We used 210 medium (5 g L^−1^ sucrose, 8 g L^–1^ casein enzymatic hydrolysate, 4 g L^−1^ yeast extract, 3 g L^−1^ K_2_HPO4 and 0.3 g L^−1^ MgSO_4_·7H_2_O, pH 7.0) to prepare for electro-component cell production and the TGM medium (10 g L^−1^ tryptone, 5 g L^−1^ yeast extract, 20 g L^−1^ glycerol, 10 g L^−1^ glucose, 0.7 g L^–1^ K_2_HPO_4_, and 0.25 g L^−1^ MgSO_4_.7H_2_O) for the EPS production assay. *Escherichia coli* (*E*. *coli*) DH5α cells were used as a host for the construction of all recombinant vectors. The *E*. *coli* BL21(DE3) strain was used as a host for expressing recombinant proteins with the pET30a, pET22b or pET28b vectors (Novagen, USA), and the *E*. *coli* TB1 strain was used as a host for expressing recombinant proteins with the pMAL-p2X vector (Novagen). Appropriate antibiotics were added to the media when needed at the following concentrations: kanamycin (50 μg ml^–1^), spectinomycin (150 μg ml^–1^) and ampicillin (100 μg ml^–1^). Electro-competent cells were prepared by washing bacterial cells thoroughly three times with ice-cold glycerol (10%). The transformation conditions of bacterial cells were set at 1.8 kV cm^–1^, 25 μF and 200 Ω and conducted in a Bio-Rad Pulser XCell (Bio-Rad, USA).

### Construction of mutant and genetic complementation

General molecular biology experiments, including PCR, DNA ligation, enzyme restriction and western blotting, followed the protocols from Molecular Cloning unless otherwise mentioned. All in-frame deletion (markerless) mutants were constructed using the suicide vector pK18mobsacB by a homologous, double crossover method. Briefly, the 5′ and 3′ genomic sequences of a targeted region were amplified using the primers listed in S2 Table, and correct PCR products were ligated into pK18mobsacB. Point mutants were constructed from the corresponding deletion mutants using pK18mobsacB with the point mutated genes. The recombinant pK18mobsacB vector was electroporated into *X*. *campestris* pv. *campestris* competent cells to generate single-crossover mutants by selection on NYG plates containing kanamycin. Next, single-crossover mutants were cultured in NYG medium for 1–2 h and then grown on NYG plates containing 10% sucrose to select second-round homologous crossovers. Candidate bacterial mutants were verified by PCR and sequencing. To genetically complement mutants, a full-length gene with a native promoter was amplified using primers listed in S2 Table and ligated into the broad-host vector pHM2. Then, the pHM2 recombinant vector was used to complement the mutated genes. The recombinant medium-copy, broad-host-range vector pBBR1MCS2 that carries full-length sequences of the genes of interest (under the control of the P*lac* promoter) was used to construct overexpression strains.

### Semi-quantitative western blotting

Briefly, bacterial culture grown to OD_600 nm_ = 0.5 and 0.1 ml or 1.0 ml cell cultures were transferred to into two new tubes, A and B, respectively. The tube B were centrifuged for 10 min at 4°C at 5,000 g. Cell pellets in tube B were resuspended in 80 μl ddH_2_O and 20 μl 5×sample loading buffer and the mixtures were boiled for 15 min before being subjected to12% (w/v) SDS-PAGE gels for electrophoresis analysis. Proteins were then electrotransferred onto a Fluoro Trans polyvinylidene fluoride membrane (PALL, USA) following the manufacturer's protocol and were detected with immunoblotting by the antiserum against RavS, RavA or RavR and HRP-conjugate goat anti-rabbit IgG secondary antibody (Abmart, China) at a dilution of 1:5,000. The protein bands were visualized by Enhanced ECL Chemiluminescent Substrate Kit (Yeasen, China). The densities of protein bands were determined by Image J. The amounts of individual protein bands were calculated from the standard curves derived from a set of purified and quantified RavS, RavA or RavR protein standards run on the same blot. Protein levels were normalised to bacterial cells, which were counted in a serially diluted bacterial culture from tube A that was grown on NYG plates for 48 hours. To estimate the concentration of a protein in a cell, morphology of 30 bacterial cells were selected and their volumes were calculated as cylinders. Each experiment was performed with three biological replicates.

### Virulence assays

Plant inoculation and virulence assays were conducted using six-week-old cabbage cultivar *Brassica oleracea* cv. Zhonggan 11 as host plants. The WT strain of *X*. *campestris* pv. *campestris* ATCC 33913 and sterile 10 mM MgCl_2_ were used as positive and negative controls, respectively. All bacterial strains were cultured overnight in NYG medium containing appropriate antibiotics. Cells were collected and washed with 10 mM MgCl_2_ and the concentrations were adjusted to OD_600_ = 0.1 before inoculation using sterile scissors. After inoculation, the plants were kept in a greenhouse at 25–30°C and relative humidity >80%. The lesion length was measured 10–12 d after inoculation. For each strain, at least 30 cutting sites were made to evaluate the virulence level.

### EPS production assays

An assay of EPS production was conducted according to a previous study with small modifications [37, 54]. Bacterial strains were cultured at 28°C in NYG medium until the OD_600_ = 0.8. Then, 200 μL subculture was inoculated into 20 mL TGM medium and cultured at 28°C for 72 h before measurement. To determine the production of EPS, the supernatants of 12 mL bacterial cultures were separated by centrifugation at 25,000 *g* for 30 min. A total of 24 mL ethanol and 1.2 mL saturated KCl were added into the supernatants and the mixtures were kept at 4°C for 4 h. The precipitated EPS was pelleted by centrifugation at 25,000 *g* for 30 min and washed twice with 12 mL 95% ethanol. The bacterial cells and EPS were dried at 60°C overnight before determination of their dry weights. EPS production was quantified by the ratio of the weight of EPS vs. the dry weight of bacterial cells.

### Swimming motility assays

NYG semi-soft agar (0.15% agar) motility plates were used to determine the swimming capability of bacterial strains. Semi-soft plates were prepared and allowed to cool and dry at room temperature for 3 h prior to inoculation. Strains were cultured at 28°C overnight and adjusted to OD_600_ = 0.8. Then, 2.5 μL of the mixture was transferred to the motility plates and incubated at 28°C for 28 h. The plates were photographed and the diameter of the migration zone of bacteria was measured. Assays were performed with at least three biological replicates, each containing 10 repeats. Statistical analysis was performed using the two-tailed Student’s *t-*test.

### Negative staining and transmission electron microscopy (TEM)

Bacterial strains were cultured at 28°C for 36 h. Negative staining and TEM observations were performed. Briefly, bacterial cells were acquired from NYG plates and gently suspended in 40 μL ddH_2_O for 15 min. The bacterial turbid liquid was then spotted onto 400 mesh carbon-coated copper grids, which were glow discharged for 15 s immediately prior to use. Samples on the grids were negatively stained twice with 0.15% uranyl acetate before blotting with filter paper. Pictures were taken with a JEM-1400 electron microscope (JEOL, Japan) at an operating voltage of 80 kV.

### Flagellated cell ratio statistics and flagellar length measurements

The number of flagellated cells (*N*_f_) and non-flagellar cells (*N*_nf_) in the TEM images were counted and the flagellated cell ratio = *N*_f_/(*N*_f_ + *N*_nf_) determined. Assays were performed with three biological replicates and the number of counted cells in each assay was larger than 100. Statistical analysis was performed using the two-tailed Student’s *t-*test.

To measure flagellar length, the tagged image file (TIF) format pictures of cells were transformed to the AutoCAD Drawing Database (DWG) format and then opened by auto Desk auto CAD 2014 software. Flagella were traced by the PLINE model and the relative lengths were calculated by the PROPERTIES model. The scale segment of each picture was measured in the same way to calculate the flagellar length. Assays were repeated with three biological replicates and the number of measured flagella in each assay was larger than 30.

### qRT-PCR

qRT-PCR was used to measure the level of mRNA. Total RNA was extracted by TRIzol (Invitrogen, USA). The DNA contamination in total RNA samples was eliminated by RNAase-free DNase I (Ambion, USA). The first strand of cDNA was generated using random primers (Promega, USA) and Superscript III reverse transcriptase (Invitrogen). qRT-PCR was conducted using Maxima SYBR Green (Fermentas, USA) in a DNA Engine Option 2 System (Bio-Rad), according to the manufacturer’s instructions. Amplification of tmRNA was used as an internal control. Generally, a qRT-PCR experiment was repeated independently three times with three technical repeats of each sample. A representative of all the biological repeats was selected and reported.

### Protein expression, purification and polyclonal antibodies

C-terminal, His_6_-tagged recombinant proteins were produced by corresponding recombinant pET30a, pET22b or pET28b (Novagen) vectors that were transformed into the *E*. *coli* BL21(DE3) strain. His_6_-tagged proteins were extracted and purified using affinity chromatography with Ni-NTA agarose beads (Novagen), according to the manufacturer′s instructions. N-terminal MBP-tagged recombinant proteins were produced by the corresponding recombinant pMAL-P2X (Novagen) vector that was transformed into the *E*. *coli* TB1 strain. If necessary, the TEV protease cleavage sequence was appended at the N-terminus of the target protein. The MBP fusion protein was purified and the elute buffer was exchanged to the reaction buffer (50 mM Tris-HCl, pH 8.0, 50 mM NaCl, 0.5 mM EDTA and 1 mM DTT), then incubated with TEV protease at a ratio of 1:100 for 8 h at 4°C. Cleaved MBP or uncleaved fusion protein was removed by MBP resin and the TEV protease was removed by Ni-NTA agarose beads. Purified target protein was obtained by size exclusion chromatography with a Superdex 75 10/300 GL column (GE Healthcare, Piscataway, NJ, USA). Purified proteins were concentrated using Centricon YM-10 columns (Millipore) and the elute buffer was changed to the storage buffer (50 mM Tris-HCl, pH 8.0, 0.5 mM EDTA, 50 mM NaCl and 5% glycerol) for further use or storage at −80°C. Protein preparations were examined for purity by SDS-PAGE and quantified by a Bradford assay (Bio-Rad). Polyclonal antiserums of RavR, RavS and RavA were prepared by immunising rabbits with approximately 3 mg purified, soluble proteins. The polyclonal antibodies of RpfC and HPPK were reported by our previous studies [32, 55].

### Gel filtration chromatography

The purified EAL protein was concentrated by VivaSpin Turbo, 5K MWCO (Sartorius, German). A total of 15 μM protein and 300 μM c-di-GMP are incubated in phosphodiesterase reaction buffer (50 mM Tris-HCl pH 7.5, 250 mM NaCl, 25 mM KCl, 10 mM MgCl_2_ and 2 mM DTT) at 4°C for 30 min and subsequently subjected to gel filtration with Fast Protein Liquid Chromatography AKTA Purifier 10 with Frac-900 (GE Healthcare, USA). The ATKA system was pre-equilibrated with phosphodiesterase reaction buffer at a flow rate of 0.5 ml/min and then applied to a Superdex 75 10/300 GL column to separate dimer and monomer. The elution profiles were collected at A_280_ and confirmed by SDS-PAGE and Coomassie brilliant blue staining.

### *In vitro* autophosphorylation and the phosphoryl transfer assay

An *in vitro* autophosphorylation assay was conducted as described in our previous study [32, 55]. Purified protein was incubated with 10 μM ATP containing 10 μCi [γ-^32^P]ATP (PerkinElmer, USA) in the reaction buffer (50 mM Tris-HCl, pH 7.8, 25 mM NaCl, 25 mM KCl, 5 mM MgCl_2_, 2 mM DTT) for the indicated time at 28°C. If necessary, c-di-GMP was added to the mixture at the same time as ATP. For the phosphoryl transfer assay, purified RR protein or its derivative was added into the reaction mixture containing the phosphorylated HK. The autophosphorylation or phosphoryl transfer reaction was terminated by adding 5× SDS-PAGE loading buffer. Phosphorylated proteins were separated by 12% SDS-PAGE. After electrophoresis, gels were exposed to a phosphor screen (GE Healthcare) and the autoradiographic signals were detected by a Typhoon FLA7000 (GE Healthcare).

### Phos-tag acrylamide gel analysis

Phos-tag acrylamide gels were prepared according to the instructions described by the manufacturer with minor modifications. Phos-tag acrylamide running gels contained 8% or 12% (w/v) 29:1 acrylamide:N, N”-methylene-bis-acrylamide, 375 mM Tris pH 8.8, 0.1% (w/v) SDS. All the gels were copolymerized with 50 μM Phos-tag acrylamide and 100 μM MnCl_2_. All stacking gels contained 5% (w/v) 29:1 acrylamide:N, N”-methylene-bis-acrylamide, 125 mM Tris pH 6.8, 0.1% (w/v) SDS. All Phostag acrylamide containing gels were run at 4°C for 3.5 hours under constant voltage (150 V). Coomassie brilliant blue staining was used to detect the phosphorylated proteins.

### Microscale thermophoresis assay

The interaction between c-di-GMP nucleotides and various proteins was measured by using MST with a fluorescein-labelled c-di-GMP (2′-Fluo-AHC-c-di-GMP, abbreviated as fl-c-di-GMP; 2′-Fluo-AHC-c-di-AMP, fl-c-di-AMP; 2′-Fluo-AHC-cGMP, fl-cGMP; Biolog, Germany). These chemicals contain carboxyfluorescein that has excitation and emission wavelengths of 497 nm and 520 nm, respectively, which can be detected directly by a MST instrument. The experiments were performed on a Monolith NT.115 device using standard treated capillaries (NanoTemper Technologies, Germany). The concentration of the particular protein varied from 0.036 to 150 μM with a 2-fold gradient and the concentration of fl-c-di-GMP was constant at 20 nM. Fluorescence intensity due to thermophoresis was recorded using the blue channel optics of the instrument (*λ*_ex_ = 470 ± 15 nm, *λ*_em_ = 520 ± 10 nm) during a 30 s period of infrared laser heating at 80% of the maximum laser power, followed by a 5 s cooling period. Measurements were performed in buffer containing 20 mM HEPES, pH 7.5, 150 mM NaCl, 2 mM MgCl_2_, 2 mM DTT and 0.05% Tween 20. The KD Fit of NanoTemper Analysis software (ver. 1.5.41) was used for fitting the curve and calculation of the dissociation constant (*K*_d_). Assays were repeated with at least three biological replicates in triplicate.

### Thermal shift assay

Thermal shift assays were performed as previously described with minor modification[32]. Briefly, purified protein was added to the reaction to a final concentration of 5 μM in the MST buffer [20 mM HEPES, pH 7.5, 150 mM NaCl, 2 mM MgCl_2,_ 2 mM DTT, 0.05% Tween 20 and 1:500 dilution of SYPRO Orange Dye (Invitrogen, USA)]. A melt curve protocol was run on a Bio-Rad qPCR instrument (Bio-Rad, USA). The fluorescence was measured using the ROX reporter with a temperature gradient of 20–95°C in 1.0°C increments at 30 second intervals. Melt curve data were trimmed to three data points after maximum and the data were plotted with Boltzmann model to obtain the temperature midpoint of unfolding (T_mid_) of the protein using Prism 5.0 software (GraphPad). Three biological replicates were assayed in triplicate and statistical significance was determined with two-tailed Student’s *t-*test.

### Synthesis and purification of [^32^P]c-di-GMP

[^32^P]-labelled c-di-GMP was chemically synthesised using a labelled [^32^P]GTP (3000 Ci/mmol, PerkinElmer, USA) and the purified His_6_-tagged enzyme tDGC; a protein construct with a key residue mutation (R158A) of diguanylate cyclase from *Thermotoga maritime* [41]. Five micromolar tDGC and 20 μCi [^32^P]GTP (mixed with 50 μM cold GTP) were added to a mixture of 20 μL reaction buffer (300 mM NaCl, 50 mM Tris-HCl, pH 7.5, 20 mM MgCl_2_, and 2 mM DTT). After 3 h at 45°C, the reaction was terminated by heating at 98°C for 10 min. The precipitated protein was removed by centrifugation at 20,000 *g* for 5 min. Radioactive [^32^P]c-di-GMP was tested by separation on a polyethyleneimine-cellulose plate (1:1.5 (v/v) saturated (NH_4_)_2_SO_4_ and 1.5 M KH_2_PO_4_, pH 3.6). The mixture contained more than 95% [^32^P]c-di-GMP without further purification.

### In vivo c-di-GMP quantification

c-di-GMP extraction was performed as described previously with modifications [34]. Briefly, 8 mL bacterial culture grown to OD_600_ = 0.8 was centrifuged at 5000 *g* and 4°C for 10 min. Cell pellets were resuspended in 2× 800 μL NYG medium and 0.2 and 1.0 mL of the cell resuspension was transferred to two tubes, A and B, respectively. The tubes were centrifuged at 5,000 *g* and 4°C for 10 min. The cell pellet in tube B was resuspended in 300 μL extraction solution (40% acetonitrile, 40% methanol and 20% water), incubated on ice for 15 min and lysed by a non-contact ultrasonication system (Bioruptor UCD-200, Diagenode, Belgium) for 10 min with an alternating 30 s power on and 30 s power off procedure. Samples were then centrifuged at 20,000 *g* and 4°C for 10 min and the supernatant was transferred into a 2.0 mL tube. Extraction was repeated twice with 200 μL extraction solution but heating at 95°C was omitted. The combined supernatant fluids of three extractions were completely evaporated. c-di-GMP powders were resuspended in 100 μL HPLC-grade ddH_2_O and analysed by liquid chromatography-tandem mass spectrometry (LC-MS/MS) on an AB SCIEX QTRAP 4500 system (AB SCIEX, USA). A Synergi Hydro-RP 80A LC column (4 μM, 150 × 2 mm, Phenomenex, Torrance, CA, USA) was used for reversed-phase liquid chromatography. Solvent A was 0.1% acetic acid in 10 mM ammonium acetate and solvent B was 0.1% formic acid in methanol. The gradient used was as follows: time (*t*) = 0–4 min, 98% solvent A, 2% solvent B; t = 10–15 min, 5% solvent A, 95% solvent B. The injection volume was 5 μL and the flow rate for chromatography was 200 μL/min. The amount of c-di-GMP in samples was calculated with a standard curve generated from pure c-di-GMP (Sigma-Aldrich, St. Louis, MO, USA) suspended in ddH_2_O (Biolog, Germany). c-di-GMP levels were normalised to bacterial cells, which were counted in a serially diluted bacterial culture in tube A that was grown on NYG plates for 48 h. Each c-di-GMP quantification experiment was performed with three biological replicates. The levels of c-di-GMP were compared with those of WT with the two-tailed Student’s *t-*test.

### Diguanyl cyclase (DGC) and phosphodiesterase (PDE) activity assay

For the DGC activity assay, purified proteins were added to the reaction buffer (300 mM NaCl, 50 mM Tris-HCl, pH 7.5, 20 mM MgCl_2_, 2 mM DTT) and 20 μCi [^32^P]GTP (mixed with 50 μM cold GTP) was added. After 1 h at 28°C, the reaction was terminated by the addition of an equal volume of 0.5 M EDTA, pH 8.0. For the PDE activity assay, the purified RavR protein was added to the reaction buffer consisting of 50 mM Tris-HCl, pH 7.5, 250 mM NaCl, 25 mM KCl, 10 mM MgCl_2_ and 2 mM DTT. Reactions were initiated by the addition of ~1 μM of [^32^P]c-di-GMP substrate. Reactions were incubated at 28°C for 30 min before termination by adding an equal volume of 0.5 M EDTA, pH 8.0. For the DGC and PDE assays, reaction products were mixed with an equal volume of running buffer consisting of 1:1.5 (v/v) saturated (NH_4_)_2_SO_4_ and 1.5 M KH_2_PO_4_, pH 3.6. Two microlitres of the reaction mixture was spotted and dried onto Cellulose PEI TLC plates (Selecto Scientific, USA). Plates were developed in running buffer, air-dried and exposed to a storage phosphor screen (GE Healthcare) and then the autoradiographic signals were recorded on a Typhoon FLA7000 (GE Healthcare).

### Homology modelling and molecular docking analyses

The three-dimensional structure of the DHp-CA domain of RavS was generated by homology modelling methods. Homology modelling was carried out using Modeller 9.10 software. The known structures of CpxAHDC (4BIU), DivL (4EW8) and VraS (4GT8) were used for multiple template homology modelling.

The structure of c-di-GMP was extracted from the c-di-GMP-VCA0042 structure complex (PDB ID: 2RDE). The structure of DHp-CA was treated by adding hydrogen atoms, calculating the charge and combining nonpolar hydrogens. Docking calculations were carried out using Autodock 4.0 software. Affinity (grid) maps of 40 × 40 × 40 Å grid points were generated using the Autogrid program. After 100 calculations, the lowest energy conformation was chosen for c-di-GMP and protein interaction analysis.

### Ethics Statement

There were no animal experiments in this study.

## Supporting information

S1 Fig Molar ratio of RavA, RavR and RavS in *X*. *campesris* pv. *campestris*. (a) Semi-quantitative western blotting test of different concentrations of purified protein and cellular RavA, RavR and RavS in *X*. *ccampestris* pv. *campestris*. Three biological replicates were performed with each comprising of three technical repeats. (b–d) Quantification of western blotting band intensities by Image J software in (a) (*n* = 3).
(PDF)

S2 Fig Inactivation of *ravS*, *ravR* and *ravA* did not impact bacterial growth, production of extracellular enzymes and the oxidative stress response. (a) Growth curves of bacterial strains. All strains were cultured overnight in NYG medium. The OD_600_ was measured at intervals of 2 h. Standard deviations are given for each data point (*n* = 3). (b–d) Production of extracellular enzymes of bacterial strains. (b) Extracellular protease. (c) Extracellular cellulase. (d) Extracellular amylase. (e) Resistance to oxidative stress. A 0.2-mL aliquot of each bacterial culture (OD_600_ = 0.8) was spread onto an NYG plate and dried at room temperature. Then 5 μL of 1.63 M H_2_O_2_ was inoculated onto a round filter paper. In (b–e) the plates were incubated at 28°C for 36 h. Average diameters of degradation or inhibition zones (*n* = 5) are shown in the figures.
(PDF)

S3 Fig mRNA levels of the flagella-related and *hrp* genes in *X*. *campesris* pv. *campestris*. (a) Genetic location of *ravARS* genes and flagella-associated genes. Arrows indicate direction of genes. (b) mRNA levels of flagella-related genes. (c) mRNA levels of *hrp* genes. (d) mRNA levels of *hrpG* gene in various strains. Transcription levels of *fliC*, *fliD*, *motA*, *motB*, *flgA*, *flgB*, *flhA flhB*, *hrpD*, *hrpF* and *hrpG* were quantified by qRT-PCR. In studying *hrp* gene expression, the bacterial cells were induced in the XVM2 medium. Three biological replicates each comprising of three repeats were performed. The average value and standard deviation are shown (*n* = 3). Asterisk indicates statistically significant (*P* ≤ 0.05), as measured by a two-tailed Student′s *t*-test.
(PDF)

S4 Fig Epistasis analysis reveals that *ravS* is downstream of *ravA*-*ravR* in regulation. Deletion of *ravS* or point mutation of the conserved phosphorylation site (*ravS*^H500A^) suppresses the mutational effects of *ravA* or *ravR* deletions. Representative pictures of virulence (a) swimming ability (b) and flagella development (c) are shown.
(PDF)

S5 Fig EAL domain of RavR has phosphodiesterase activity. (a) Synthesis of ^32^P-labelled c-di-GMP by tDGC cyclase *in vitro*. c-di-GMP was synthesized and labelled by using tDGC cyclase from [^32^P]GTP. Samples were analysed on thin-layer chromatography (TLC) plates. (b) RavR did not possess diguanylate cyclase (DGC) activity. None or 5 μM affinity purified proteins including GGDEF, RavR, RavR^ΔEAL^ or tDGC were added to the DGC reaction mixture with α-^32^P-labeled GTP. After incubation at 28°C for 60 min, samples were analysed by TLC assays. (c) Recombinant EAL-domain containing protein naturally forms homodimer. The purified EAL protein was separated by a molecular sieve and the molecular weight of each fraction was determined by analytic ultra-centrifugation. (d) Phosphorylation of RavR did not affect its PDE activity. The enzymatic reactions contained 25 μM ^32^P-labelled c-di-GMP were analysed by TLC. RavR or RavR^D496A^ (5 μM), RavA (10 μM) and ATP (2 mM) were added as indicated. (e) Intensity of the signals in (d), measured by ImageJ. (f) Intracellular c-di-GMP concentrations of various strains detected by LC-MS/MS analysis. All the experiments were repeated independently three times.
(PDF)

S6 Fig Both RavA and RavS have autokinase activity and transfer the phosphoryl group to RavR in a RavR^D496^ dependent manner. (a) RavA phosphorylates RavR *in vitro*. Autophosphorylation of the histidine kinase RavA and its recombinant derivative RavA^H164A^ in the presence of [γ-^32^P]ATP at room temperature for 10 min. RavA phosphotransfer to RavR was then carried out for 30 s. Assays contained 10 μM of soluble protein RavA or RavA^H164A^. Ten micromolar RavR or RavR^D496A^ was added into the mixtures as indicated. The experiment was repeated three times. (b) RavA phosphorylated the recombinant protein RavR^ΔEAL^ *in vitro*. Assays contained 15 μM of soluble protein RavA or RavA^H164A^. Five micromolar RavR^ΔEAL^ or RavR^ΔEAL(D496A)^ was added as indicated. (c) RavS^ΔN^ but not RavS^ΔTrM^ possesses robust autophosphorylation activity. Five micromolar of RavS^ΔTrM^ or RavS^ΔN^ was added into the *in vitro* autophosphorylation mixture in the presence of [γ-^32^P]ATP at room temperature for 10 min. (d–f) Detection phosphorylated HK and RR using Phos-tag acrylamide gel. (d) RavA phosphorylates RavR or RavR^ΔEAL^ *in vivo*. RavA or RavA^H164A^ was incubated with 2 mM ATP at 28°C for 15 min, respectively. RavR (or RavR^D496A^) or RavR^ΔEAL^ (or RavR^ΔEAL(D496A)^) was then added into mixtures for 2 min. (e and f) RavS phosphorylates RavR^ΔEAL^ or RavR^EAL-AAA^ *in vitro*. RavS^ΔN^ was autophosphorylated with 2 mM ATP at 28°C for 15 minutes. Various recombinant RavR proteins, including RavR^ΔEAL^, RavR^ΔEAL(D496A)^, RavR^EAL-AAA^ or RavR^EAL-AAA(D496A)^ were added and incubated for 30 min at 28°C. A total of 100 μM c-di-GMP were added as indicated. The reactions were stopped with 3 × SDS loading buffer and the products were separated by 8% or 12% acrylamide gels at 4°C, the gels were stained with Coomassie brilliant blue (d–f). Each experiment was repeated three times.
(PDF)

S7 Fig c-di-GMP specifically binds to RavS to enhance the RavS-RavR phosphotransfer. (a) Expression of truncated RavS and RavR proteins. Numbers indicate the sites of amino acid residues of RavS or RavR. (b) RavS^ΔN^ specifically interacts with fl-c-di-GMP. The interactions of RavS^ΔN^, RavA, VgrS and RavR^ΔEAL^ with fl-c-di-GMP were measured by microscale thermophoresis (MST). Twenty nanomolar fluorescein-labelled nucleotides and increasing concentrations of proteins were co-incubated and potential interactions were measured by MST. The dissociation constant (*K*_d_) was determined to estimate the binding affinity. Averages and standard deviations are shown (*n* = 3). (c) Time course analyses of the impact of c-di-GMP on the velocity of RavS-RavR phosphotransfer. Representative pictures in Fig 5B are shown. Each experiment was repeated three times.
(PDF)

S8 Fig Substitution of Arg^656^ residue did not affect the stability of the recombinant RavS protein *in vitro* and *in vivo*. (a), R656A substitution did not affect the thermal stability of recombinant DHp-CA protein of RavS. Thermal stability of DHp-CA or DHP-CA^R656A^ was tested using Thermal shift assay (TSA). Three independent experiments were performed and a representative result is shown. The average and the standard deviation of three midpoints of melt curves were displayed. (b), R656A substitution did not affect the autokinase activity of RavS^ΔN^. RavS^ΔN^ or RavS^ΔN(R656A)^ autophosphorylates in the presence of [γ-^32^P]ATP at room temperature for various minutes. In vitro phosphorylation assay was used to measure the autokinase activity. The experiment was repeated for three times. (c), *ravS*^R656A^ point mutation did not affect the stability of RavS in cell. Western blotting was used to detect the RavS. Samples were separated on 8% SDS-PAGE gel. Each experiment was repeated independently three times.
(PDF)

S9 Fig Overexpression of *ravS* (OE-*ravS*) but not *ravS*^H500A^ rescues the swimming motility of the ravR^ΔEAL^ mutant. (a) Overexpression of *ravS* in bacterial cells. RavS was detected by western blotting. RNAP was used as the loading control. The experiment was repeated three times. (b–i) Phenotypic characterization of the effect of *ravS* overexpression. WT-EV (harbouring a medium-copy, broad-host pBBR1MCS2 vector), ravR^ΔEAL^ΔravS-EV, ravR^ΔEAL^ΔravS::OE-ravS and ravR^ΔEAL^ΔravS::OE-ravS^H500A^ strains of *X*. *campestris* pv. *campestris*. (b–c) Bacterial virulence. Bacterial strains were inoculated onto plant leaves of *Brassica olercaeae* cv Zhonggan 11. The lesion length was recorded 10 d after inoculation (*n* = 30). (d) Production of extracellular polysaccharides (EPS). Bacterial strains were grown in TGM medium at 28°C for 72 h before EPS quantification, which was calculated as the dry weight of EPS vs. the dry weight of bacterial cells (*n* = 3). (e) Swimming motility. Bacterial strains were inoculated in NYG plates containing 0.15% agar and grown below 28°C for 28 h. Average diameters of the migration zones were measured (*n* = 10). (f) Flagella of bacterial strains. Bacterial flagella were observed by transmission electron microscopy (TEM) after negative staining. Representative images of each strain are shown. Upper panel: bacteria population profile; lower panel: a single bacterium. (g) Ratio of bacterial cells with flagella. For each strain, cells with flagella were counted (*n* = 100). (h) Flagellar length of bacterial strains (*n* = 30). (i) *fliC* mRNA level in bacterial strains. The amount of *fliC* mRNA was measured by qRT-PCR. Amplification of cDNA of tmRNA was used as the internal control. In (c–e) and (g–i), vertical bars represent standard deviations; asterisk: significant differences were tested by Student’s *t*-test (*P* ≤ 0.05). The experiment was repeated three times and a representative result is shown.
(PDF)

S1 Table Bacterial strains and plasmids used in this study. (PDF)

S2 Table Primers used in this study. (PDF)
