# Supplementary material for: Cyclic-di-GMP binds to histidine kinase RavS to control RavS-RavR phosphotransfer and regulates the bacterial lifestyle transition between virulence and swimming
Source: PLoS Pathog. 2019 Aug 13;15(8):e1007952. doi: 10.1371/journal.ppat.1007952 (PMC6707612; doi:10.1371/journal.ppat.1007952)
Supplement: S1 Fig — (a) Semi-quantitative western blotting test of different concentrations of purified protein and cellular RavA, RavR and RavS in X. ccampestris pv. campestris. Three biological replicates were performed with each comprising of three technical repeats. (b–d) Quantification of western blotting band intensities by Image J software in (a) (n = 3). (PDF) [file ppat.1007952.s001.pdf]

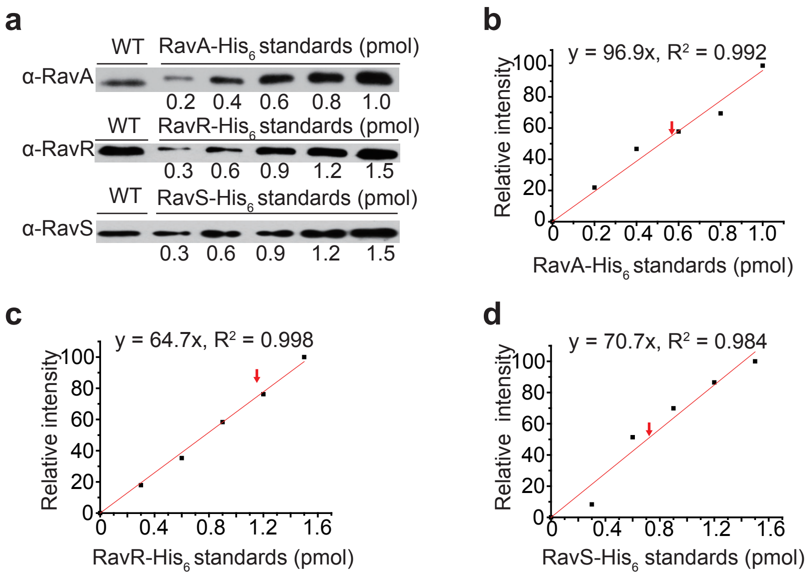

S1 Fig. Molar ratio of RavA, RavR and RavS in *X. campesris pv. campestris*.
(**a**) Semi-quantitative western blotting test of different concentrations of purified protein and cellular RavA, RavR and RavS in *X. ccampestris pv. campestris*. Three biological replicates were performed with each comprising of three technical repeats. (**b–d**) Quantification of western blotting band intensities by Image J software in (**a**) (n = 3).
