# Supplementary material for: Cyclic-di-GMP binds to histidine kinase RavS to control RavS-RavR phosphotransfer and regulates the bacterial lifestyle transition between virulence and swimming
Source: PLoS Pathog. 2019 Aug 13;15(8):e1007952. doi: 10.1371/journal.ppat.1007952 (PMC6707612; doi:10.1371/journal.ppat.1007952)
Supplement: S2 Fig — (a) Growth curves of bacterial strains. All strains were cultured overnight in NYG medium. The OD600 was measured at intervals of 2 h. Standard deviations are given for each data point (n = 3). (b–d) Production of extracellular enzymes of bacterial strains. (b) Extracellular protease. (c) Extracellular cellulase. (d) Extracellular amylase. (e) Resistance to oxidative stress. A 0.2-mL aliquot of each bacterial culture (OD600 = 0.8) was spread onto an NYG plate and dried at room temperature. Then 5 μL of 1.63 M H2O2 was inoculated onto a round filter paper. In (b–e) the plates were incubated at 28°C for 36 h. Average diameters of degradation or inhibition zones (n = 5) are shown in the figures. (PDF) [file ppat.1007952.s002.pdf]

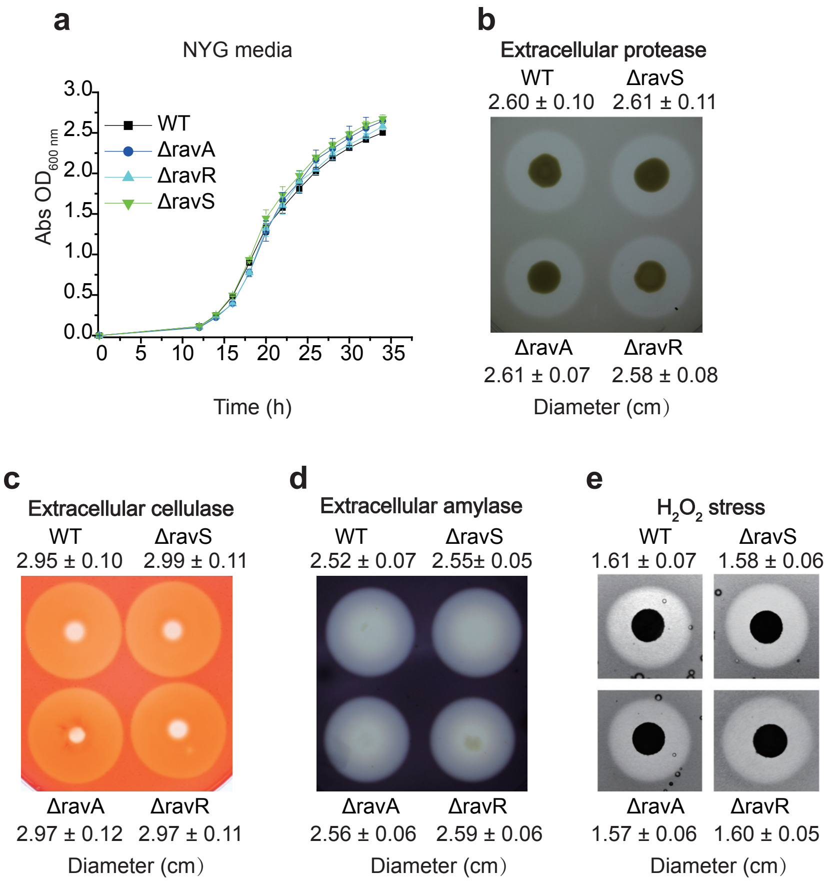

S2 Fig. Inactivation of *ravS*, *ravR* and *ravA* did not impact bacterial growth, production of extracellular enzymes and the oxidative stress response. (**a**) Growth curves of bacterial strains. All strains were cultured overnight in NYG medium. The OD_{600} was measured at intervals of 2 h. Standard deviations are given for each data point (n = 3). (**b–d**) Production of extracellular enzymes of bacterial strains. (**b**) Extracellular protease. (**c**) Extracellular cellulase. (**d**) Extracellular amylase. (**e**) Resistance to oxidative stress. A 0.2-mL aliquot of each bacterial culture (OD_{600} = 0.8) was spread onto an NYG plate and dried at room temperature. Then 5 μL of 1.63 M H_2O_2 was inoculated onto a round filter paper. In (**b–e**) the plates were incubated at 28 °C for 36 h. Average diameters of degradation or inhibition zones (n = 5) are shown in the figures.
