# Supplementary material for: Cyclic-di-GMP binds to histidine kinase RavS to control RavS-RavR phosphotransfer and regulates the bacterial lifestyle transition between virulence and swimming
Source: PLoS Pathog. 2019 Aug 13;15(8):e1007952. doi: 10.1371/journal.ppat.1007952 (PMC6707612; doi:10.1371/journal.ppat.1007952)
Supplement: S3 Fig — (a) Genetic location of ravARS genes and flagella-associated genes. Arrows indicate direction of genes. (b) mRNA levels of flagella-related genes. (c) mRNA levels of hrp genes. (d) mRNA levels of hrpG gene in various strains. Transcription levels of fliC, fliD, motA, motB, flgA, flgB, flhA flhB, hrpD, hrpF and hrpG were quantified by qRT-PCR. In studying hrp gene expression, the bacterial cells were induced in the XVM2 medium. Three biological replicates each comprising of three repeats were performed. The average value and standard deviation are shown (n = 3). Asterisk indicates statistically significant (P ≤ 0.05), as measured by a two-tailed Student′s t-test. (PDF) [file ppat.1007952.s003.pdf]

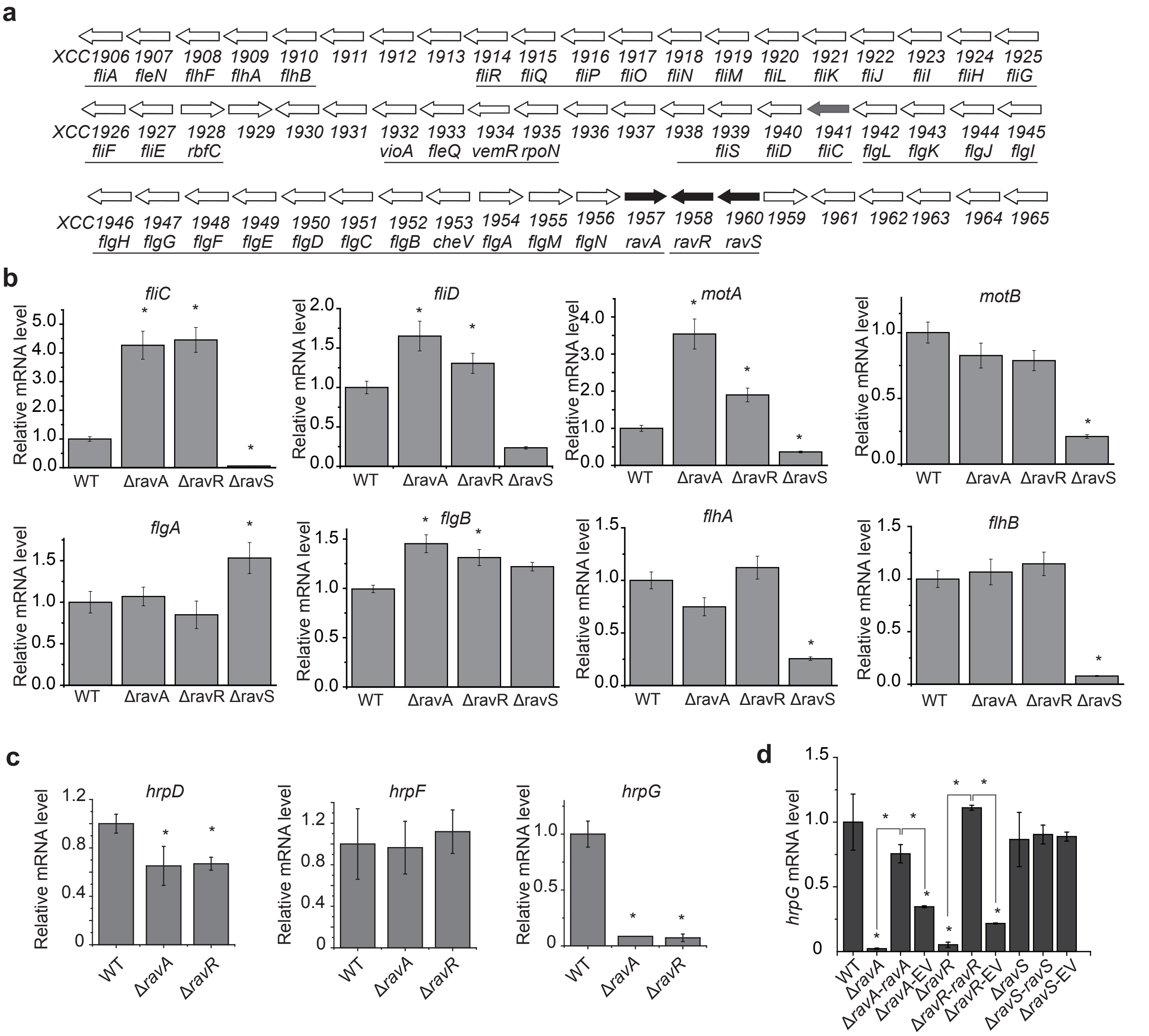

S3 Fig. mRNA levels of the flagella-related and *hrp* genes in *X. campesris pv. campestris*.

(**a**) Genetic location of *ravARS* genes and flagella-associated genes. Arrows indicate direction of genes. (**b**) mRNA levels of flagella-related genes. (**c**) mRNA levels of hrp genes. (**d**) mRNA levels of *hrpG* gene in various strains. Transcription levels of *fliC*, *fliD*, *motA*, *motB*, *flgA*, *flgB*, *flhA*, *flhB*, *hrpD*, *hrpF* and *hrpG* were quantified by qRT-PCR. In studying *hrp* gene expression, the bacterial cells were induced in the XVM2 medium. Three biological replicates each comprising of three repeats were performed. The average value and standard deviation are shown (n = 3). Asterisk indicates statistically significant (P ≤ 0.05), as measured by a two-tailed Student's *t*-test.
