# Supplementary material for: Cyclic-di-GMP binds to histidine kinase RavS to control RavS-RavR phosphotransfer and regulates the bacterial lifestyle transition between virulence and swimming
Source: PLoS Pathog. 2019 Aug 13;15(8):e1007952. doi: 10.1371/journal.ppat.1007952 (PMC6707612; doi:10.1371/journal.ppat.1007952)
Supplement: S4 Fig — Deletion of ravS or point mutation of the conserved phosphorylation site (ravSH500A) suppresses the mutational effects of ravA or ravR deletions. Representative pictures of virulence (a) swimming ability (b) and flagella development (c) are shown. (PDF) [file ppat.1007952.s004.pdf]

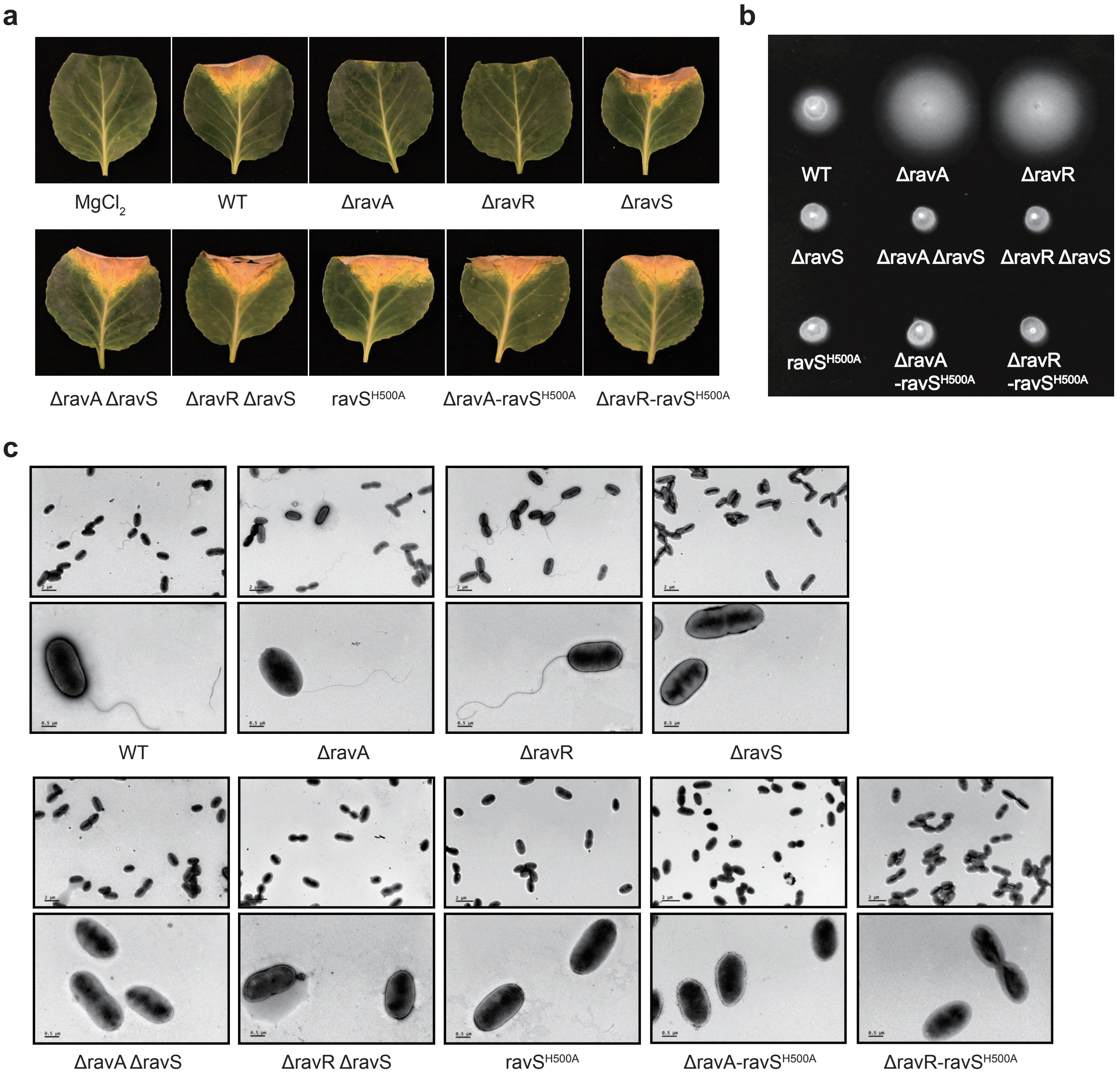

S4 Fig. Epistasis analysis reveals that *ravS* is downstream of *ravA-ravR* in regulation.
Deletion of *ravS* or point mutation of the conserved phosphorylation site (*ravS*^{H500A}) suppresses the mutational effects of *ravA* or *ravR* deletions. Representative pictures of virulence (**a**) swimming ability (**b**) and flagella development (**c**) are shown.
