# Supplementary material for: Cyclic-di-GMP binds to histidine kinase RavS to control RavS-RavR phosphotransfer and regulates the bacterial lifestyle transition between virulence and swimming
Source: PLoS Pathog. 2019 Aug 13;15(8):e1007952. doi: 10.1371/journal.ppat.1007952 (PMC6707612; doi:10.1371/journal.ppat.1007952)
Supplement: S5 Fig — (a) Synthesis of 32P-labelled c-di-GMP by tDGC cyclase in vitro. c-di-GMP was synthesized and labelled by using tDGC cyclase from [32P]GTP. Samples were analysed on thin-layer chromatography (TLC) plates. (b) RavR did not possess diguanylate cyclase (DGC) activity. None or 5 μM affinity purified proteins including GGDEF, RavR, RavRΔEAL or tDGC were added to the DGC reaction mixture with α-32P-labeled GTP. After incubation at 28°C for 60 min, samples were analysed by TLC assays. (c) Recombinant EAL-domain containing protein naturally forms homodimer. The purified EAL protein was separated by a molecular sieve and the molecular weight of each fraction was determined by analytic ultra-centrifugation. (d) Phosphorylation of RavR did not affect its PDE activity. The enzymatic reactions contained 25 μM 32P-labelled c-di-GMP were analysed by TLC. RavR or RavRD496A (5 μM), RavA (10 μM) and ATP (2 mM) were added as indicated. (e) Intensity of the signals in (d), measured by ImageJ. (f) Intracellular c-di-GMP concentrations of various strains detected by LC-MS/MS analysis. All the experiments were repeated independently three times. (PDF) [file ppat.1007952.s005.pdf]

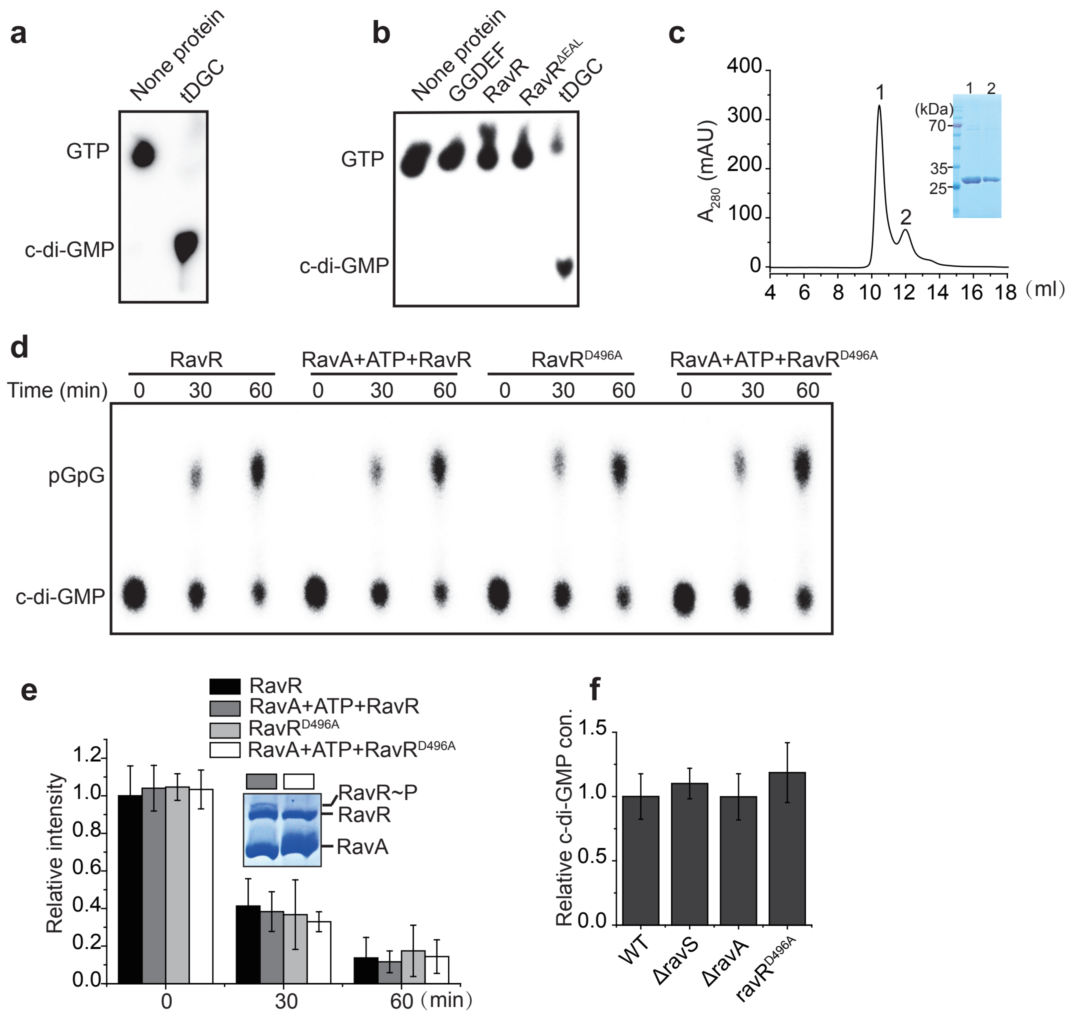

S5 Fig. EAL domain of RavR has phosphodiesterase activity. (a) Synthesis of 32P-labelled c-di-GMP by tDGC cyclase in vitro. c-di-GMP was synthesized and labelled by using tDGC cyclase from [^{32}P]GTP. Samples were analysed on thin-layer chromatography (TLC) plates. (b) RavR did not possess diguanylate cyclase (DGC) activity. None or 5 μM affinity purified proteins including GGDEF, RavR, RavR$^{\Delta EAL}$ or tDGC were added to the DGC reaction mixture with α-^{32}P-labeled GTP. After incubation at 28 °C for 60 min, samples were analysed by TLC assays.
(c) Recombinant EAL-domain containing protein naturally forms homodimer. The purified EAL protein was separated by a molecular sieve and the molecular weight of each fraction was determined by analytic ultra-centrifugation.
(d) Phosphorylation of RavR did not affect its PDE activity. The enzymatic reactions contained 25 μM ^{32}P-labelled c-di-GMP were analysed by TLC. RavR or RavR^{D496A} (5 μM), RavA (10 μM) and ATP (2 mM) were added as indicated.
(e) Intensity of the signals in (d), measured by ImageJ. (f) Intracellular c-di-GMP concentrations of various strains detected by LC-MS/MS analysis. All the experiments were repeated independently
three times.
