# Supplementary material for: Cyclic-di-GMP binds to histidine kinase RavS to control RavS-RavR phosphotransfer and regulates the bacterial lifestyle transition between virulence and swimming
Source: PLoS Pathog. 2019 Aug 13;15(8):e1007952. doi: 10.1371/journal.ppat.1007952 (PMC6707612; doi:10.1371/journal.ppat.1007952)
Supplement: S6 Fig — (a) RavA phosphorylates RavR in vitro. Autophosphorylation of the histidine kinase RavA and its recombinant derivative RavAH164A in the presence of [γ-32P]ATP at room temperature for 10 min. RavA phosphotransfer to RavR was then carried out for 30 s. Assays contained 10 μM of soluble protein RavA or RavAH164A. Ten micromolar RavR or RavRD496A was added into the mixtures as indicated. The experiment was repeated three times. (b) RavA phosphorylated the recombinant protein RavRΔEAL in vitro. Assays contained 15 μM of soluble protein RavA or RavAH164A. Five micromolar RavRΔEAL or RavRΔEAL(D496A) was added as indicated. (c) RavSΔN but not RavSΔTrM possesses robust autophosphorylation activity. Five micromolar of RavSΔTrM or RavSΔN was added into the in vitro autophosphorylation mixture in the presence of [γ-32P]ATP at room temperature for 10 min. (d–f) Detection phosphorylated HK and RR using Phos-tag acrylamide gel. (d) RavA phosphorylates RavR or RavRΔEAL in vivo. RavA or RavAH164A was incubated with 2 mM ATP at 28°C for 15 min, respectively. RavR (or RavRD496A) or RavRΔEAL (or RavRΔEAL(D496A)) was then added into mixtures for 2 min. (e and f) RavS phosphorylates RavRΔEAL or RavREAL-AAA in vitro. RavSΔN was autophosphorylated with 2 mM ATP at 28°C for 15 minutes. Various recombinant RavR proteins, including RavRΔEAL, RavRΔEAL(D496A), RavREAL-AAA or RavREAL-AAA(D496A) were added and incubated for 30 min at 28°C. A total of 100 μM c-di-GMP were added as indicated. The reactions were stopped with 3 × SDS loading buffer and the products were separated by 8% or 12% acrylamide gels at 4°C, the gels were stained with Coomassie brilliant blue (d–f). Each experiment was repeated three times. (PDF) [file ppat.1007952.s006.pdf]

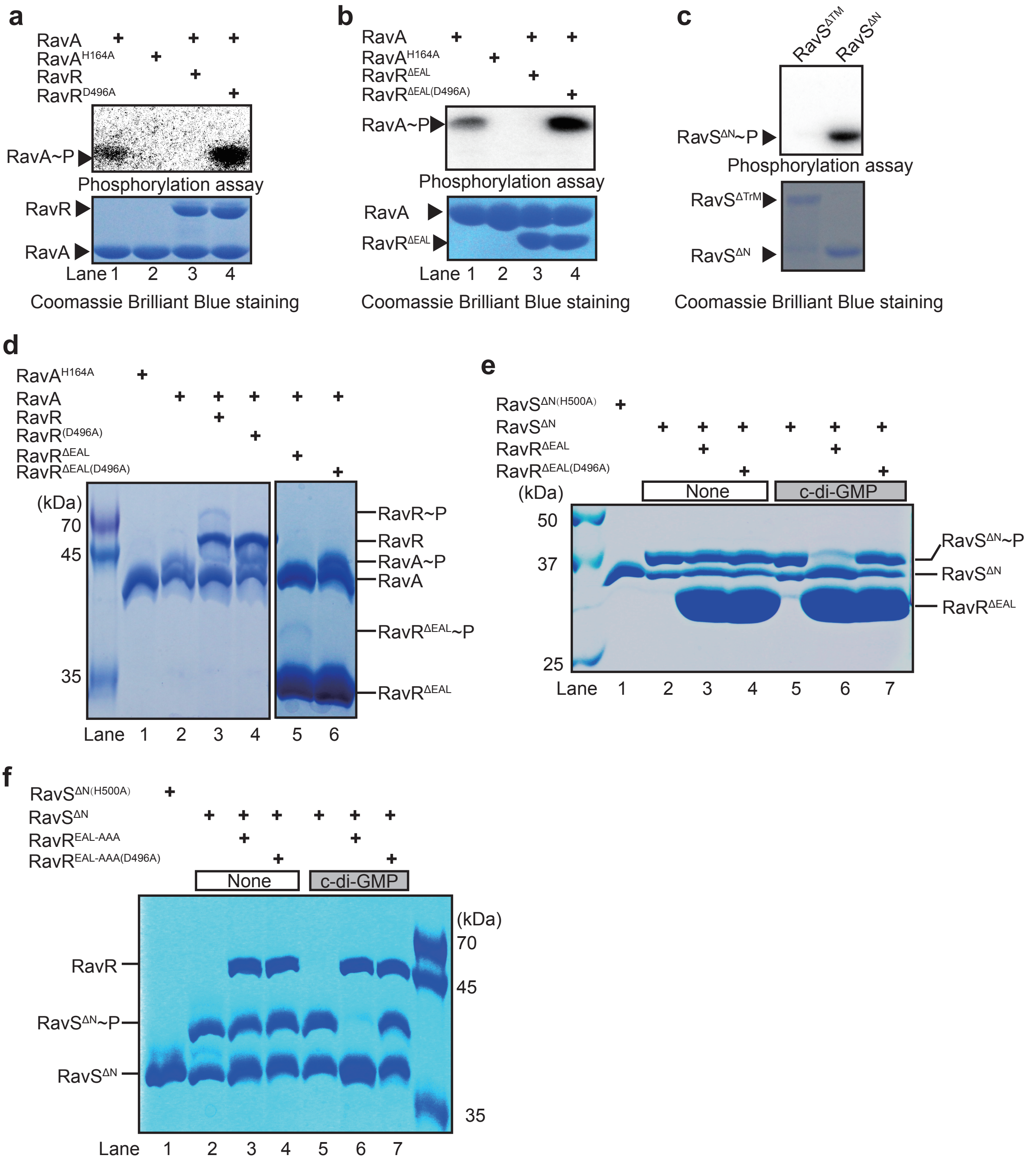

S6 Fig. Both RavA and RavS have autokinase activity and transfer the phosphoryl group to RavR in a $RavR^{D496}$ dependent manner. (a) RavA phosphorylates RavR in vitro. Autophosphorylation of the histidine kinase RavA and its recombinant derivative $RavA^{H164A}$ in the presence of [γ-^{32}P]ATP at room temperature for 10 min. RavA phosphotransfer to RavR was then carried out for 30 s. Assays contained 10 μM of soluble protein RavA or $RavA^{H164A}$. Ten micromolar RavR or $RavR^{D496A}$ was added into the mixtures as indicated. The experiment was repeated three times. (b) RavA phosphorylated the recombinant protein $RavR^{\Delta EAL}$ in vitro. Assays contained 15 μM of soluble protein RavA or $RavA^{H164A}$. Five micromolar $RavR^{\Delta EAL}$ or $RavR^{\Delta EAL(D496A)}$ was added as indicated. (c) $RavS^{\Delta N}$ but not $RavS^{\Delta TrM}$ possesses robust autophosphorylation activity. Five micromolar of $RavS^{\Delta TrM}$ or $RavS^{\Delta N}$ was added into the in vitro autophosphorylation mixture in the presence of [γ-^{32}P]ATP at room temperature for 10 min. (d–f) Detection phosphorylated HK and RR using Phos-tag acrylamide gel. (d) RavA phosphorylates RavR or $RavR^{\Delta EAL}$ in vivo. RavA or $RavA^{H164A}$ was incubated with 2 mM ATP at 28 °C for 15 min, respectively. RavR (or $RavR^{D496A}$) or $RavR^{\Delta EAL}$ (or $RavR^{\Delta EAL(D496A)}$) was then added into mixtures for 2 min. (e – f) $RavS^{\Delta N}$ phosphorylates $RavR^{\Delta EAL}$ or $RavR^{EAL\text{-}AAA}$ in vitro. $RavS^{\Delta N}$ was autophosphorylated with 2 mM ATP at 28 °C for 15 min. A total of 20 μM various recombinant RavR proteins, including $RavR^{\Delta EAL}$, $RavR^{\Delta EAL(D496A)}$, $RavR^{EAL\text{-}AAA}$ or $RavR^{EAL\text{-}AAA(D496A)}$ were added and incubated for 30 min at 28°C. A total of 100 μM c-di-GMP were added as indicated. The reactions were stopped with 3 × SDS loading buffer and the products were separated by 8% or 12% acrylamide gels at 4 °C, the gels were stained with Coomassie brilliant blue (d–f). Each experiment was repeated three times.
