# Supplementary material for: Cyclic-di-GMP binds to histidine kinase RavS to control RavS-RavR phosphotransfer and regulates the bacterial lifestyle transition between virulence and swimming
Source: PLoS Pathog. 2019 Aug 13;15(8):e1007952. doi: 10.1371/journal.ppat.1007952 (PMC6707612; doi:10.1371/journal.ppat.1007952)
Supplement: S7 Fig — (a) Expression of truncated RavS and RavR proteins. Numbers indicate the sites of amino acid residues of RavS or RavR. (b) RavSΔN specifically interacts with fl-c-di-GMP. The interactions of RavSΔN, RavA, VgrS and RavRΔEAL with fl-c-di-GMP were measured by microscale thermophoresis (MST). Twenty nanomolar fluorescein-labelled nucleotides and increasing concentrations of proteins were co-incubated and potential interactions were measured by MST. The dissociation constant (Kd) was determined to estimate the binding affinity. Averages and standard deviations are shown (n = 3). (c) Time course analyses of the impact of c-di-GMP on the velocity of RavS-RavR phosphotransfer. Representative pictures in Fig 5B are shown. Each experiment was repeated three times. (PDF) [file ppat.1007952.s007.pdf]

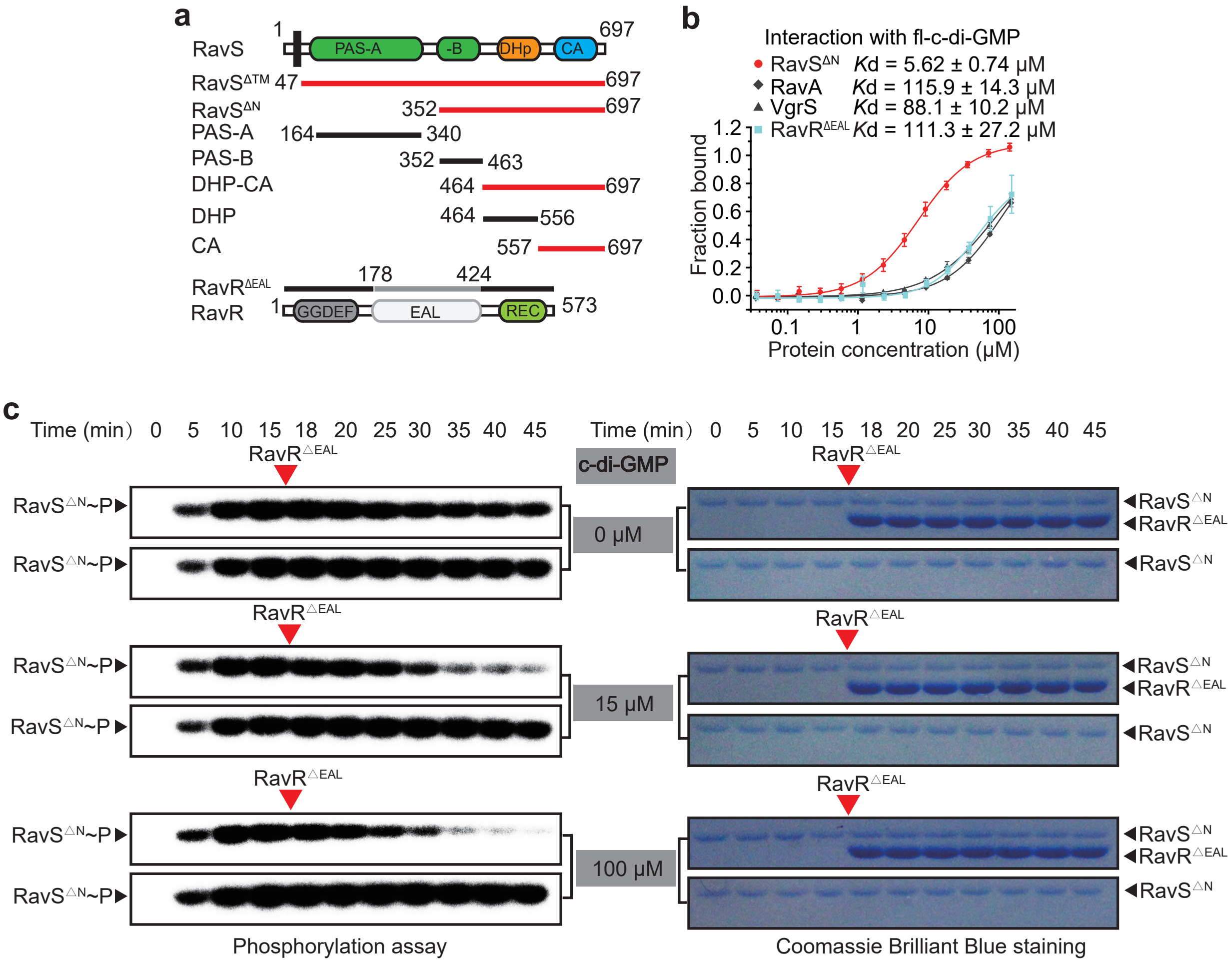

S7 Fig. c-di-GMP specifically binds to RavS to enhance the RavS-RavR phosphotransfer. (a) Expression of truncated RavS and RavR proteins. Numbers indicate the sites of amino acid residues of RavS or RavR. (b) RavS$^{\Delta N}$ specifically interacts with fl-c-di-GMP. The interactions of RavS$^{\Delta N}$, RavA, VgrS and RavR$^{\Delta EAL}$ with fl-c-di-GMP were measured by microscale thermophoresis (MST). Twenty nanomolar fluorescein-labelled nucleotide and increasing concentrations of proteins were co-incubated and potential interactions were measured by MST. The dissociation constant (*K*d) was determined to estimate the binding affinity. Averages and standard deviations are shown (n = 3). (c) Time course analyses of the impact of c-di-GMP on the velocity of RavS-RavR phosphotransfer. Representative pictures in Fig 5b are shown.Each experiment was repeated three times.
