# Supplementary material for: Cyclic-di-GMP binds to histidine kinase RavS to control RavS-RavR phosphotransfer and regulates the bacterial lifestyle transition between virulence and swimming
Source: PLoS Pathog. 2019 Aug 13;15(8):e1007952. doi: 10.1371/journal.ppat.1007952 (PMC6707612; doi:10.1371/journal.ppat.1007952)
Supplement: S8 Fig — (a), R656A substitution did not affect the thermal stability of recombinant DHp-CA protein of RavS. Thermal stability of DHp-CA or DHP-CAR656A was tested using Thermal shift assay (TSA). Three independent experiments were performed and a representative result is shown. The average and the standard deviation of three midpoints of melt curves were displayed. (b), R656A substitution did not affect the autokinase activity of RavSΔN. RavSΔN or RavSΔN(R656A) autophosphorylates in the presence of [γ-32P]ATP at room temperature for various minutes. In vitro phosphorylation assay was used to measure the autokinase activity. The experiment was repeated for three times. (c), ravSR656A point mutation did not affect the stability of RavS in cell. Western blotting was used to detect the RavS. Samples were separated on 8% SDS-PAGE gel. Each experiment was repeated independently three times. (PDF) [file ppat.1007952.s008.pdf]

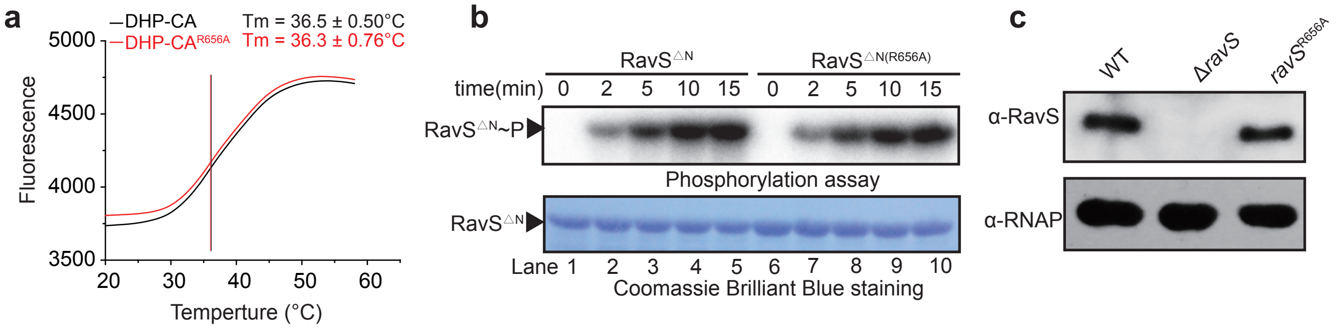

S8 Fig. Substitution of Arg^{656} residue did not affect the stability of the recombinant RavS protein *in vitro* and *in vivo*. (a), R656A substitution did not affect the thermal stability of recombinant DHp-CA protein of RavS. Thermal stability of DHp-CA or DHP-CA^{R656A} was tested using Thermal shift assay (TSA). Three independent experiments were performed and a representative result is shown. The average and the standard deviation of three midpoints of melt curves were displayed. (b), R656A substitution did not affect the autokinase activity of RavS$^{\Delta N}$. RavS$^{\Delta N}$ or RavS$^{\Delta N(R656A)}$ autophosphorylates in the presence of [γ-^{32}P]ATP at room temperature for various minutes. *In vitro* phosphorylation assay was used to measure the autokinase activity. The experiment was repeated for three times. (c), ravS^{R656A} point mutation did not affect the stability of RavS in cell. Western blotting was used to detect the RavS. Samples were separated on 8% SDS-PAGE gel. Each experiment was repeated independently three times.
