# Supplementary material for: Cyclic-di-GMP binds to histidine kinase RavS to control RavS-RavR phosphotransfer and regulates the bacterial lifestyle transition between virulence and swimming
Source: PLoS Pathog. 2019 Aug 13;15(8):e1007952. doi: 10.1371/journal.ppat.1007952 (PMC6707612; doi:10.1371/journal.ppat.1007952)
Supplement: S9 Fig — (a) Overexpression of ravS in bacterial cells. RavS was detected by western blotting. RNAP was used as the loading control. The experiment was repeated three times. (b–i) Phenotypic characterization of the effect of ravS overexpression. WT-EV (harbouring a medium-copy, broad-host pBBR1MCS2 vector), ravRΔEALΔravS-EV, ravRΔEALΔravS::OE-ravS and ravRΔEALΔravS::OE-ravSH500A strains of X. campestris pv. campestris. (b–c) Bacterial virulence. Bacterial strains were inoculated onto plant leaves of Brassica olercaeae cv Zhonggan 11. The lesion length was recorded 10 d after inoculation (n = 30). (d) Production of extracellular polysaccharides (EPS). Bacterial strains were grown in TGM medium at 28°C for 72 h before EPS quantification, which was calculated as the dry weight of EPS vs. the dry weight of bacterial cells (n = 3). (e) Swimming motility. Bacterial strains were inoculated in NYG plates containing 0.15% agar and grown below 28°C for 28 h. Average diameters of the migration zones were measured (n = 10). (f) Flagella of bacterial strains. Bacterial flagella were observed by transmission electron microscopy (TEM) after negative staining. Representative images of each strain are shown. Upper panel: bacteria population profile; lower panel: a single bacterium. (g) Ratio of bacterial cells with flagella. For each strain, cells with flagella were counted (n = 100). (h) Flagellar length of bacterial strains (n = 30). (i) fliC mRNA level in bacterial strains. The amount of fliC mRNA was measured by qRT-PCR. Amplification of cDNA of tmRNA was used as the internal control. In (c–e) and (g–i), vertical bars represent standard deviations; asterisk: significant differences were tested by Student’s t-test (P ≤ 0.05). The experiment was repeated three times and a representative result is shown. (PDF) [file ppat.1007952.s009.pdf]

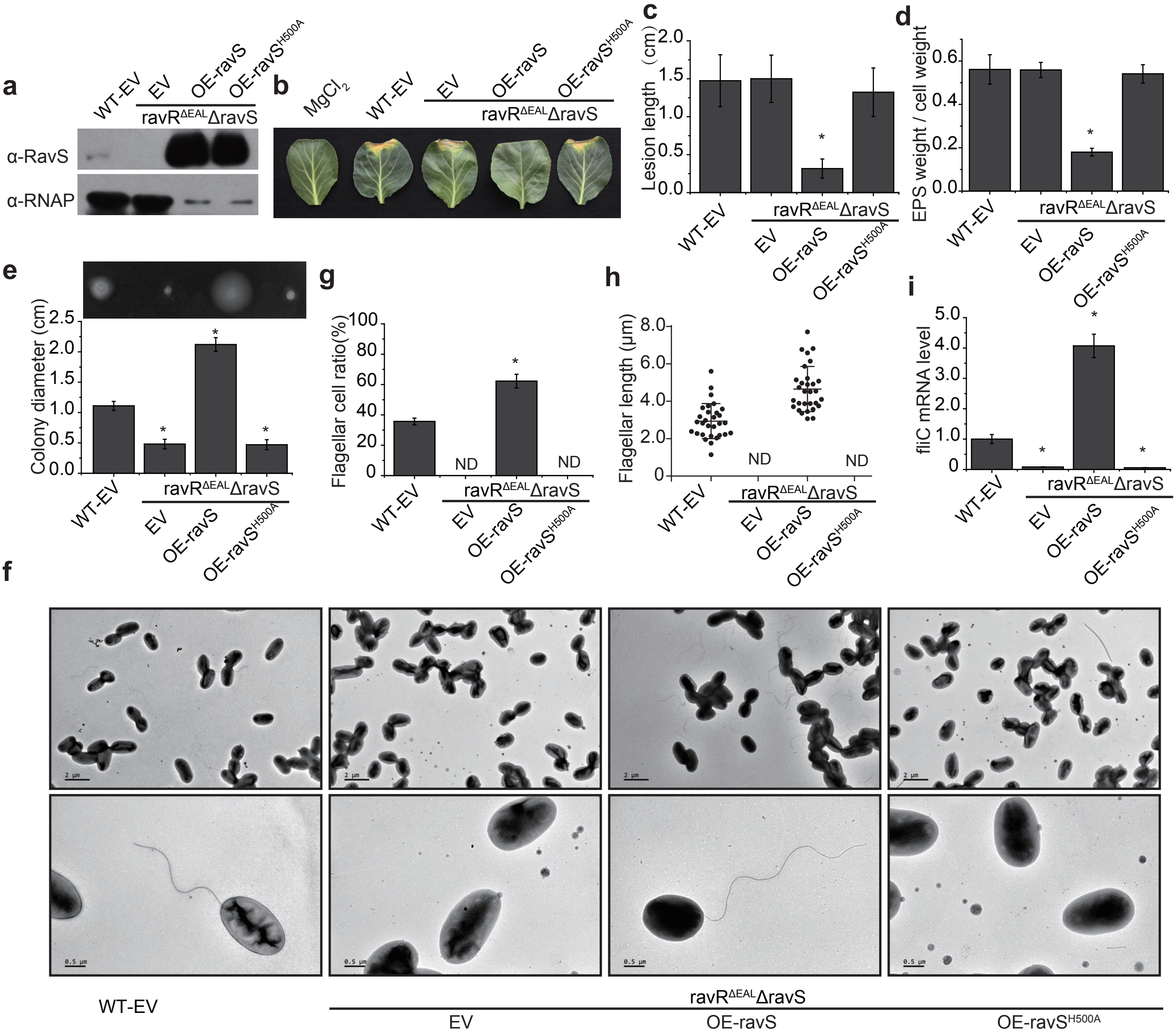

S9 Fig. Overexpression *ravS* (OE-*ravS*) but not *ravS*^{H500A} rescues the swimming motility of the *ravR*^{ΔEAL} mutant.(**a**) Overexpression of *ravS* in bacterial cells. RavS was detected by Western blotting, RNAP was used as loading control. The experiment was repeated three times.(**b–i**) Phenotypic characterization of the effect of *ravS* overexpression. WT-EV (harboring a medium copy, broad-host pBBR1MCS2 vector), *ravR*^{ΔEAL}ΔravS-EV, ravR^{ΔEAL}ΔravS::OE-*ravS*, ravR^{ΔEAL}ΔravS::OE-*ravS*^{H500A} strains of *X. campestris* pv. *campestris*. (**b–c)** Bacterial virulence. Bacterial strains were inoculated onto plant leaves of *Brassica olercaeae* cv Zhonggan 11. Lesion length was recorded 10 days after inoculation (n = 30). (**d**) Production of extracellular polysaccharides (EPS). Bacterial strains were grown in TGM medium at 28 °C for 72 hours before EPS quantification, which was calculated as the dry weight of EPS vs. the dry weight of bacterial cells (n = 3). (**e)** Swimming motility. Bacterial strains were inoculated in NYG plates containing 0.15% agar and grew under 28 °C for 28 h. Average diameters of the migration zones were measured (*n* = 10). (**f)** Flagella of bacterial strains. Bacterial flagella were observed by transmission electron microscopy (TEM) after negative staining. Representative images of each strain are shown. Upper panel: bacteria population profile; Lower panel: a single bacterium. (**g**) Ratio of bacterial cells with flagella. For each strain, cells with flagella were counted (*n* =100). (**h**) Flagellar length of bacterial strains (*n* = 30). (**i**) *fliC* mRNA level in bacterial strains. The amount of *fliC* mRNA was measured by qRT-PCR. Amplification of cDNA of tmRNA was used as internal control. In (**c–e**), (**g–i**), vertical bar indicates standard deviation; asterisk: significant difference, tested by Student's *t*-test (P ≤ 0.05) were measured. The experiment was repeated three times and a representative result is shown.
