# Supplementary material for: Cyclic-di-GMP binds to histidine kinase RavS to control RavS-RavR phosphotransfer and regulates the bacterial lifestyle transition between virulence and swimming
Source: PLoS Pathog. 2019 Aug 13;15(8):e1007952. doi: 10.1371/journal.ppat.1007952 (PMC6707612; doi:10.1371/journal.ppat.1007952)
Supplement: S1 Table — (PDF) [file ppat.1007952.s010.pdf]

S1 Table | Bacterial strains and plasmids used in this study.

Strains	Genotype or properties*	Source/ Reference
**Strains**		
***Escherichia coli* strains**		
DH5α	*FhuA2Δ(argF-lacZ)U169 phoA glnV44 Φ80Δ(lacZ)M15 gyrA96 recA1 endA1 thi-1 hsdR17*	Lab collection
BL21(DE3)	*E.coli B* F^- *dcm ompT hsd*(r_{B-} m_{B-})*gal λ(DE3)*	Novagen
TB1	*ara Δ（lac_proAB） rpsL （_Φ80_ lacΔZM15） hsdR*	BioLabs
***Xanthomonas campestris* pv. *campestris* strains**		
WT	Wild-type of *X.campestris* pv.*campestris* ATCC 33913	Lab collection
M0300	ΔravA *, XCC1957* (*ravA*) in-frame deletion mutant	This study
M0301	ΔravA-EV, ΔravA strain containing a blank pHM2 vector, Sp^r	This study
M0302	ΔravA-ravA, ΔravA strain containing a recombinant vector of pHM2::P*ravA-ravA*. Sp^r	This st, udy
M0303	ΔravR *, XCC1958* (*ravR*) in-frame deletion mutant	This study
M0304	ΔravR-EV, ΔravR strain containing a blank pHM2 vector, Sp^r	This study
M0305	ΔravR-ravR, ΔravR strain containing a recombinant vector of pHM2::P*ravS-ravR*. Sp^r	This study
M0306	ΔravS *, XCC1960* (*ravS*) in-frame deletion mutant	This study
M0307	ΔravS-EV, ΔravS strain containing a blank pHM2 vector, Sp^r	This study
M0308	ΔravS-ravS, ΔravS strain containing a recombinant vector of pHM2::P*ravS-ravS*. Sp^r	This study
M0309	ΔravAΔravS*,* double mutant of *ravA* and *ravS*	This study
M0310	ΔravRΔravS, double mutant of *ravR* and *ravS*	This study
M0311	$ravS^{H500A}$, a *ravS* point mutation with the coding sequence of His^{500} being substituted by that of Ala	This study
M0312	ΔravA-$ravS^{H500A}$, double mutant of *ravA* and *ravS*^{H500A}	This study
M0313	ΔravR- $ravS^{H500A}$, double mutant of *ravR* and *ravS*^{H500A}	This study
M0314	$ravR^{\Delta EAL}$, *ravR* with EAL domain in-frame deletion mutant	This study
M0315	$ravR^{D496A}$, a *ravR* point mutation with the coding sequence of Asp^{496} being substituted by that of Ala	This study
M0316	$ravR^{\Delta EAL(D496A)}$, double mutant of *ravR*$^{\Delta EAL}$ and *ravR*^{D496A}	This study
M0317	$ravR^{D496A}$-$ravS^{H500A}$, double mutant of *ravR*^{D496A} and *ravS*^{H500A}	This study

M0318	ravR$^{\Delta EAL(D496A)}$ -ravS^{H500A}, triple mutant of *ravR*$^{\Delta EAL}$, *ravR*$^{\Delta EAL(D496A)}$ and ravS^{H500A}	This study
M0319	ravS^{R656A}, a *ravS* point mutation with the coding sequence of Arg^{656} being substituted by that of Ala	This study
M0320	ravR$^{\Delta EAL}$ -ravS^{R656A}, double mutant of *ravR*$^{\Delta EAL}$ and *ravS*^{R656A}	This study
M0321	ravS$^{R656A\text{-}H500A}$, double mutant of *ravS*^{R656A} and *rav*$^{\text{-}H500A}$	This study
M0322	ravR$^{\Delta EAL}$ -ravS$^{R656A\text{-}H500A}$, triple mutant of *ravR*$^{\Delta EAL}$, *ravS*^{R656A} and *ravS* ^{H500A}	This study
M0323	ravR$^{\Delta EAL}$ -ΔravS-EV, M0341 strain containing a blank vector pBBR1M CS2. Kan^{r}	This study
M0324	ravR$^{\Delta EAL}$ -ΔravS::OE-ravS, M0341 strain containing a recombinant vector pBBR1M CS2::*ravS*, overexpression of *ravS* in M0341 strain. Kan^{r}	This study
M0325	ravR$^{\Delta EAL}$ -ΔravS::OE-ravS^{H500A}, M0341 strain containing a recombinant vector pBBR1MCS2::*ravS*^{H500A}, overexpression of *ravS*^{H500A} in M0341 strain. Kan^{r}	This study
**Plasmids**		
pK18mobsacB	Suicide plasmid for *X. campestris* pv. *campestris*, Kan^{r}	(Schafer *et al.*,1994)
pHM2	Complementary vector with no promoter before MCS, Sp^{r}	Lab collection
pBBR1MCS2	Broad-host-range vector used for genetic complementation; Kan^{r}	Lab collection
pET30a	Protein expression vector, Kan^{r}	Novagen
pET22b	Protein expression vector, Amp^{r}	Novagen
pET28b	Protein expression vector, Kan^{r}	Novagen
pMal-p2X	Protein expression vector, Amp^{r}	BioLabs
pET-RavA	pET30a::*ravA*, Kan^{r}, expressing full-length RavA	This study
pET-RavA^{H164A}	pET30a:: *ravA*^{H164A}, Kan^{r}, expressing full-length RavA with His^{164} substituted by that of Ala	This study
pET-RavR	pET30a::*ravR*, Kan^{r}, expressing full-length RavR	This study
pET-RavR^{D496A}	pET30a:: *ravR*^{D496A}, Kan^{r}, expressing full-length RavR with with Asp^{496} substituted by that of Ala	This study
pET-RavS$^{\Delta TM}$	pET30a:: *ravS*$^{\Delta TM}$ Kan^{r}, expressing cytosolic RavS (D47-R697), without transmembrane domain	This study
pET-RavS$^{\Delta N}$	pET30a:: *ravS*$^{\Delta PAS\text{-}A}$, Kan^{r}, expressing cytosolic RavS (E352-R697), without N-terminal transmembrane domain and PAS-A domain	This study
pET-DHp-CA	pET30a::DHp-CA, Kan^{r}, expressing RavS containing DHp-CA domain (E464-R697)	This study

pET-PAS-A	pET30a::*PAS-A*, Kan^r, expressing RavS PAS-A domain(T164-R340)	This study
pET-PAS-B	pET30a::*PAS-B*, Kan^r, expressing RavS PAS-B domain(E352-P463)	This study
pET-DHP	pET30a::*DHp*, Kan^r, expressing RavS DHp domain(E464-L556)	This study
pMal-CA	pMal-p2X::*CA*, Amp^r, expressing RavS CA domain (D557-R697) with TEV protease cleavage sequence at the N-terminal of CA.	This study
pET-TEV	pET22b::TEV, Amp^r, expressing TEV protease	Dr. Jin-Lan Gao
pET-DHp-CA^{S636A}	pET30a::*DHp-CA*^{S636A}, Kan^r, expressing RavS containing DHp and CA domains, with the Ser^{636} substituted by that of Ala	This study
pET-DHp-CA^{D637A}	pET30a::*DHp-CA*^{D637A}, Kan^r, expressing RavS containing DHp and CA domains, with the Asp^{637} substituted by that of Ala	This study
pET-DHp-CA^{D638A}	pET30a::*DHp-CA*^{D638A}, Kan^r, expressing RavS containing DHp and CA domains, with the Asp^{638} substituted by that of Ala	This study
pET-DHp-CA^{G639A}	pET30a::*DHp-CA*^{G639A}, Kan^r, expressing RavS containing DHp and CA domains, with the Gly^{639} substituted by that of Ala	This study
pET-DHp-CA^{D640A}	pET30a::*DHp-CA*^{D640A}, Kan^r, expressing RavS containing DHp and CA domains, with the Asp^{640} substituted by that of Ala	This study
pET-DHp-CA^{L652A}	pET30a::*DHp-CA*^{L652A}, Kan^r, expressing RavS containing DHp and CA domains, with the Leu^{652} substituted by that of Ala	This study
pET-DHp-CA^{I653A}	pET30a::*DHp-CA*^{I653A}, Kan^r, expressing RavS containing DHp and CA domains, with the Ile^{653} substituted by that of Ala	This study
pET-DHp-CA^{R656A}	pET30a:: *DHp-CA*^{R656A}, Kan^r, expressing RavS containing DHp and CA domains, with the Arg^{656} substituted by that of Ala	This study
pET-DHp-CA^{E657A}	pET30a:: *DHp-CA*^{E657A}, Kan^r, expressing RavS containing DHp and CA domains, with the Glu^{657} substituted by that of Ala	This study
pET-DHp-CA^{L666A}	pET30a:: *DHp-CA*^{L666A}, Kan^r, expressing RavS containing DHp and CA domains, with the Leu^{666} substituted by that of Ala	This study
pET-EAL	pET30a::*EAL*, Kan^r, expressing RavR EAL domain	This study
pET-$RavR^{\Delta EAL}$	pET30a:: *ravR*$^{\Delta EAL}$, Kan^r, expressing RavR protein	This study

	without EAL domain	
pET-RavR$^{\Delta EAL-(D496A)}$	pET30a:: *ravR*$^{\Delta EAL(D496A)}$, Kan^r, expressing RavR protein with D496A point mutation, without EAL domain	This study
pET-tDGC	Protein expression vector, pET28b:: tDGC, Kan^r, expressing tDGC protein with R87A point mutation	From Dr. Zhaoxun-Liang
pKS-*ravA*	pK18mobSacB::*ravA*, recombinant suicide vector for constructing ΔravA strain, Kan^r	This study
pKS-*ravR*	pK18mobSacB::*ravR*, recombinant suicide vector for constructing ΔravR strain, Kan^r	This study
pKS-*ravR*$^{\Delta EAL}$	pK18mobSacB:: *ravR*$^{\Delta EAL}$, recombinant suicide vector for constructing *ravR*$^{\Delta EAL}$ strain, Kan^r	This study
pKS-*ravR*^{D496A}	pK18mobSacB:: *ravR*^{D496A}, recombinant suicide vector for constructing *ravR*^{D496A} strain, Kan^r	This study
pKS-*ravS*	pK18mobSacB::*ravS*, recombinant suicide vector for constructing ΔravS strain, Kan^r	This study
pKS-*ravS*^{H500A}	pK18mobSacB:: *ravS*^{H500A}, recombinant suicide vector for constructing *ravS*^{H500A} strain, Kan^r	This study
pKS-*ravS*^{R656A}	pK18mobSacB:: *ravS*^{R656A}, recombinant suicide vector for constructing *ravS*^{R656A} strain, Kan^r	This study
pKS-*ravS*$^{R656A-H500A}$	pK18mobSacB:: *ravS*$^{R656A-H500A}$, recombinant suicide vector for constructing *ravS*$^{R656A-H500A}$ strain, Kan^r	This study
pHM2-*ravA*	pHM2::P_{ravA}-*ravA*, recombinant vector for genetic complementation of *ravA* mutant,Sp^r	This study
pHM2-*ravR*	pHM2::P_{ravS}-*ravR,* recombinant vector for genetic complementation of *ravR* mutant,Sp^r	This study
pHM2-*ravS*	pHM2::P_{ravS}-*ravS,* recombinant vector for genetic complementation of *ravS* mutant,Sp^r	This study
pBBR-ravS	pBBR1MCS2::ravS, recombinant vector for overexpression of ravS in ravR$^{\Delta EAL}$-ΔravS strain	This study
pBBR-ravS^{H500A}	pBBR1MCS2::ravS^{H500A}, recombinant vector for overexpression of ravS^{H500A} in ravR$^{\Delta EAL}$-ΔravS strain	This study

* Kan^r, kanamysin resistance; Sp^r,,) Spectinomycin resistance; Amp^r , Ampicillin resistance
