# Supplementary material for: Cyclic-di-GMP binds to histidine kinase RavS to control RavS-RavR phosphotransfer and regulates the bacterial lifestyle transition between virulence and swimming
Source: PLoS Pathog. 2019 Aug 13;15(8):e1007952. doi: 10.1371/journal.ppat.1007952 (PMC6707612; doi:10.1371/journal.ppat.1007952)
Supplement: S2 Table — (PDF) [file ppat.1007952.s011.pdf]

S2 Table | Primers used in this study.

Name	Sequence (forward/reverse)	Length/Description
Δ*ravA*	A:GAATTCCAAGATGCTCTACCCCGAC<br>B: AAGCTTAGCTTCCGGATCGTGTTG<br>C: AAGCTTGAGAAAGAACTTGGCAGT<br>D: GTCGACAAGTGCAGATGCCCGAAG	AB 403 bp; CD 301 bp; For pKS-*ravA* construction
Δ*ravR*	A: GGATCCCTGACCAATGCGTTGCGG<br>B: AAGCTTCGGGATCACAGGGCTCCA<br>C: AAGCTT CATGATGAGTTGCGGAAC<br>D: GTCGACGGCATCATCAACAAGCAC	AB 300 bp; CD 303 bp; For pKS-*ravR* construction
Δ*ravS*	A: GGATCCCATTGCCCGCTCCATGAG<br>B: AAGCTTGTGTTCTTCCACGATCTG<br>C: AAGCTTCTGATCGTGATGCGTGAG<br>D: GTCGACCGATATGGAAGATCATCA	AB 299 bp; CD 277 bp; For pKS-*ravS* construction
ravR$^{\Delta EAL}$	A: GGATCCGCACGGTAGCGACGAAAT<br>B: GGTACCATGCGCATTGGTGGCCGA<br>C: GGTACCGCCGGCATGGCGCTCCGC<br>D: TCGACGTGTCGGGGTAGAGCATC	AB 315 bp; CD 275 bp; For pKS-*ravR*$^{\Delta EAL}$ construction
ravR^{D496A}	A: GGATCCCTGACCAATGCGTTGCGG<br>B: GTCGACGGCATCATCAACAAGCAC<br>C: AGGTGATCCTCTCCGCCCAGCGCATGA<br>D: GCGGAGAGGATCACCTGCACATCGTTG	AB 2274 bp; For pKS-ravR^{D496A} construction
ravS^{H500A}	A: GGATCC CATTGCCCGCTCCATGAG<br>B: GTCGAC CGATATGGAAGATCATCA<br>C: CATCTCGCGCGACCTGGCCGACGACATC<br>D: GCCAGGTCGCGCGAGATGGCGCGGCGTTC	AB 2262 bp; For pKS-*ravS*^{H500A} construction
C-*ravA*	A: GTCGACCGAAGTGCAGATGCCCGA<br>B: GGTACCCTACCGCGGCTGCCGCAC	AB 1509 bp; For pHM2 –*ravA* construction
C-*ravR*	A:GAATTCTCGCTGCTTTCCAGCACTTC<br>B:TGAGGTCGTCCGCGGACCATGTGGAGCCCTGTGATC<br>C: ATGGTCCGCGGACGACCTCA<br>D: GGTACCCTATTCCCGGCCGTTCCG	AB 334 bp; CD 1722 For pHM2 –*ravR* construction
C-*ravS*	A:GAATTCTCGCTGCTTTCCAGCACTTC<br>B GGTACCTCAACGTCCACCATCTTC	AB 2410 bp; For pHM2 –*ravS* construction
OX-*ravS*	A: AAGCTTATGCACCAACACGTTGCC<br>B: GAGCTCTCAACGTCCACCATCTTCAG	AB:2094 bp; For pBBR-*ravS* or pBBR-ravS^{H500A} construction
RT-*fliC*	A: GCTTCACCACGCAGATCCG<br>B: TGGTGGCGTTGGCTGACTGC	AB 144 bp:For qRT-PCR analysis of *fliC*
RT-*fliD*	A: CGAGAACGCCAACAACG<br>B: GATCGCTTTACGGTCGTCG	AB 142 bp:For qRT-PCR analysis of *fliD*

RT-*flgA*	A: GGAATCGGGAATCGTCTGAC<br>B: ATCGGGAGTGGGGAATCG	AB 123 bp: For qRT-PCR analysis of *flgA*
RT-*flgB*	A: CGAAGGCAATCTACCGACCG<br>B: GCCTCGCCGGTCAGCTTG	AB 135 bp:For qRT-PCR analysis of *flgB*
RT-*flhA*	A: CGATGAACGCCCGCCGC<br>B: GAACAGCGCATCCAGCACC	AB 122 bp:For qRT-PCR analysis of *flhA*
RT-*flhB*	A: CGAACGCACCGAACTACCC<br>B: CTCAGCGCGGTCTTCATCC	AB 175 bp:For qRT-PCR analysis of *flhB*
RT-*motA*	A: CATCGTCGTCATCGTCAGTG<br>B: ATCTTGCCTGGCGTGCTC	AB 138 bp:For qRT-PCR analysis of *motA*
RT-*motB*	A: GTTCGCACAGCACGGGTTC<br>B: CGTGCGGCCGTCATCTC	AB 153 bp:For qRT-PCR analysis of *motB*
RT-*tmRNA*	A: GCAGCTTTCCTCGTTAATCC<br>B: CATGCTTATCCCTCCGTTAG	For qRT-PCR analysis of *tmRNA*
RavA	A: CATATGACTGCCAAGTTCTTTCTC<br>B: CTCGAGCCGCGGCTGCCGCACCG	AB 1227 bp; For pET-RavA construction
RavR	A: CATATGTGGAGCCCTGTGATC<br>B: CTCGAGTTCCCGGCCGTTCCGCAA	AB 1722 bp; For pET-RavR construction
$RavS^{\Delta TM}$	A: CATATGAACCGCATGCTCGACCTG<br>B: CTCGAGACGTCCACCATCTTCAGG	AB 1956 bp; For pET-$RavS^{\Delta TM}$ construction
$RavA^{H164A}$	A: GCTGCCGGCGTCGCGGCTGAGATCAAT<br>B: GCCGCGACGCCGGCAGCGAGCTGGC	For pET-$RavA^{H164A}$ construction
$RavR^{D496A}$	A: AGGTGATCCTCTCCGCCCAGCGCATGA<br>B: GCGGAGAGGATCACCTGCACATCGTTG	For pET-$RavR^{D496A}$ or pKS -$ravR^{D496A}$ construction
$RavS^{H500A}$	A:CATCTCGCGCGACCTGGCC GACGACATC<br>B:GCCAGGTCGCGCGAGATGGCGCGGCGTTC	For pKS -$ravS^{H500A}$ construction
R-EAL	A: CATATGAGCGAAAGCATCATTGCG<br>B: CTCGAGGGACTCGGCCGACATCGG	AB 741bp;For pET-EAL construction
S-PAS-A	A: CATATGGCACTTTTACAAGCCAAGTTCC<br>B: CTCGAGACGTTCGGTCACGTCCTG	AB 732 bp; For pET-PAS-A construction
S-PAS-B	A: CATATGGAGCAGTTCCGCGAGCTG<br>B: CTCGAGGGGCTCGCTCAGGTCGC	AB 336 bp;For protein pET-PAS-B construction
$RavS^{\Delta N}$	A: CATATGGAGCAGTTCCGCGAGCTG<br>B: CTCGAGACGTCCACCATCTTCAGG	AB 1041 bp; For protein pET- $RavS^{\Delta N}$ construction
S-DHp-CA	A: CATATGGAGCGCATGCGCGATGCA<br>B: CTCGAGACGTCCACCATCTTCAGG	AB 702 bp;For pET-DHP-CA construction
S-DHP	A: CATATGGAGCGCATGCGCGATGCA<br>B: CTCGAGGAGCTGCGGCGGCCGCAGC	AB 279 bp;For pET- DHP construction
S-CA	A:GAATTCGAGAACCTGTACTTCCAAGGGGATGCATTGGGGCTGGAAGC<br>B: CTCGAGACGTCCACCATCTTCAGG	AB 423 bp; For pMAL-CA construction

RavS^{S636A}	A: CTTTCGCCTGGAAGTCGCCGACGATGGC<br>B: GCGACTTCCAGGCGAAAGCCGTTGCCG	For pET-DHP-CA^{S636A} construction
RavS^{D637A}	A: CGCCTGGAAGTCAGCGCCGATGGCGACG<br>B: GCGCTGACTTCCAGGCGAAAGCCGT	For pET-DHP-CA ^{D637A} construction
RavS^{D638A}	A: CTGGAAGTCAGCGACGCTGGCGACGGG<br>B: GCGTCGCTGACTTCCAGGCGAAAGCCG	For pET-DHP-CA ^{D638A} construction
RavS^{G639A}	A: GAAGTCAGCGACGATGCCGACGGGTTC<br>B: GCATCGTCGCTGACTTCCAGGCGA	For pET-DHP-CA ^{G639A} construction
RavS^{D640A}	A: GTCAGCGACGATGGCGCCGGGTTCGAA<br>B: TCGCCATCGCCGCTGACTTCCAGGCG	For pET-DHP-CA ^{D640A} construction
RavS^{L652A}	A: CCGCGTGGTTTGGGGGCGATCGTGATG<br>B: GCCCCCAAACCACGCGGACCTTCCGG	For pET-DHP-CA ^{L652A} construction
RavS^{I653A}	A: CGTGGTTTGGGGCTGGCCGTGATGCGTG<br>B: GCCAGCCCCAAACCACGCGGACCTTCC	For pET-DHP-CA ^{I653A} construction
RavS^{R656A}	A: GGGCTGATCGTGATGGCTGAGCGTGCGC<br>B: GCCATCACGATCAGCCCCAAACCACG	For pET-DHP-CA^{R656A} and pKS-*ravS*^{R656A} construction
RavS^{E657A}	A: CTGATCGTGATGCGTGCGCGTGCGCA<br>B: GCACGCATCACGATCAGCCCCAAACC	For pET-DHP-CA^{E657A} construction
RavS^{L666A}	A: ACCGTCGGCGGTGCGGCCGCGATAGAA<br>B: GCCGCACCGCCGACGGTTTGCGCACGC	For pET-DHP-CA^{L666A} construction
